# Implementation strategies to improve HIV care cascade outcomes in low‐ and middle‐income countries: a systematic review from 2014 to 2021

**DOI:** 10.1002/jia2.26263

**Published:** 2024-07-05

**Authors:** Sita Lujintanon, Ingrid Eshun‐Wilson, Noelle Le Tourneau, Laura Beres, Sheree Schwartz, Stefan Baral, Ryan Thompson, Ashley Underwood, Branson Fox, Elvin H. Geng, Christopher G. Kemp

**Affiliations:** ^1^ Department of Epidemiology Johns Hopkins Bloomberg School of Public Health Baltimore Maryland USA; ^2^ Division of Infectious Diseases Washington University School of Medicine in St. Louis St. Louis Missouri USA; ^3^ Department of International Health Johns Hopkins Bloomberg School of Public Health Baltimore Maryland USA

**Keywords:** HIV, implementation science, implementation strategies, HIV cascade, systematic review, low‐ and middle‐income countries

## Abstract

**Introduction:**

In low‐ and middle‐income countries (LMICs), which are disproportionately affected by the HIV epidemic and manage limited resources, optimized implementation strategies are needed to enhance the efficiency of the HIV response. Assessing strategy usage to date could identify research gaps and inform future implementation efforts. We conducted a systematic review to describe the features and distributions of published implementation strategies attempting to improve HIV treatment service delivery and outcomes.

**Methods:**

We searched PubMed, Embase, and CINAHL and screened abstracts and full texts published between 1 January 2014 and 27 August 2021, for English‐language studies conducted in LMICs that described the implementation of HIV intervention and reported at least one HIV care cascade outcome, ranging from HIV testing to viral suppression. Implementation strategies were inductively specified, characterized by unique combinations of actor, action and action target, and summarized based on existing implementation strategy taxonomies. All strategies included in this study were independently reviewed to ensure accuracy and consistency.

**Results:**

We identified 44,126 abstracts and reviewed 1504 full‐text manuscripts. Among 485 included studies, 83% were conducted in sub‐Saharan Africa; the rest were conducted in South‐East Asia and Western Pacific (12%), and the Americas (8%). A total of 7253 unique implementation strategies were identified, including changing health service delivery (48%) and providing capacity building and support strategies (34%). Healthcare providers and researchers led 59% and 28% of the strategies, respectively. People living with HIV and their communities (62%) and healthcare providers (38%) were common strategy targets. Strategies attempting to change governance, financial arrangements and implementation processes were rarely reported.

**Discussion:**

We identified a range of published implementation strategies that addressed HIV cascade outcomes, though some key gaps exist. We may need to expand the application of implementation strategies to ensure that all stakeholders are meaningfully involved to support equitable implementation efforts across the geographic regions and target populations, and to optimize implementation outcomes.

**Conclusions:**

Some health service delivery and capacity building and support strategies have been most commonly used to date. Future research and implementation may incorporate a more diverse range of strategies and detailed reporting on their usage to inform improved HIV responses globally.

## 1 INTRODUCTION

Among people living with HIV (PLWH) globally in 2021, an estimated 85% knew their HIV status, 75% were on antiretroviral therapy (ART) and 68% achieved viral suppression [1]. Progress in achieving the Joint United Nations Programme on HIV and AIDS (UNAIDS)’ 95‐95‐95 targets by 2025 has varied between countries, regions and populations [1]. Low‐ and middle‐income countries (LMICs) are home to the majority of PLWH: an estimated 20.6 million (55%) live in eastern and southern Africa, 5.8 million (15.4%) live in Asia and the Pacific, 4.7 million (12.5%) live in western and central Africa, 2.1 million (5.6%) live in Latin America and 1.6 million (4.2%) live in eastern Europe and central Asia [2]. Certain countries in sub‐Saharan Africa and parts of the Caribbean and Latin America still experience a generalized HIV epidemic where there is a high prevalence of HIV in the general populations, yet globally key populations that are historically marginalized disproportionately bear a heightened HIV burden [1]. People who inject drugs, female sex workers, men who have sex with men and transgender women have, respectively, 35‐, 30‐, 28‐ and 14‐times the risk of acquiring HIV when compared to reproductive‐aged adults overall [1]. Despite the rollout of highly efficacious ART since the 2000s and major global investments, research collaboration, and activism, most countries are not on track to reach the UNAIDS’ targets by 2025 [1]. Declines in financial and human resources will pose additional challenges and stall efforts to increase equitable access, uptake and quality of HIV services in LMICs. The strategic use of targeted implementation strategies could help overcome these barriers and facilitate and sustain the delivery of interventions (e.g. ART) that would improve health outcomes for PLWH in LMICs.

Implementation strategies are the activities or methods that constitute *how* the interventions (i.e. the *what*) are delivered. When chosen appropriately, implementation strategies can enhance the real‐world delivery of effective health services and interventions to achieve the desired targeted outcomes [3]. In LMICs, where the HIV burden is concentrated and resources are limited, implementation strategies are particularly needed to increase the efficiency and sustainability of HIV programmes, and narrow the service and quality gaps among those most affected by HIV. Some strategies have already been incorporated into the World Health Organization (WHO)’s HIV guidelines, including removing the CD4‐count threshold on ART initiation (i.e. treat all) and recommending differentiated services through providing people‐centred care, task sharing, decentralization and service integration [4]. In HIV‐related implementation research, strategies that enhance the implementation process have also been reported, such as strategies to create demand, change infrastructure and management, and use technology to improve the delivery of HIV interventions [5]. While there are existing and extensive implementation strategy taxonomies [6, 7, 8], and some of these strategies have already been embraced by the HIV field [9], these taxonomies emerged from high‐income settings, which may not all be applicable to LMICs. Despite the rapid growth of HIV implementation research and innovative service delivery models globally, there have been no systematic attempts to summarize the progress of implementation strategy usage in resource‐limited settings. Understanding the characteristics, breadth and overall landscape of existing HIV implementation strategies can further help to identify critical gaps in research and inform future investigation and service delivery.

To further this understanding, we describe the features and distributions of implementation strategies used in the delivery of HIV interventions aiming to improve the HIV care cascade outcomes in LMICs from studies published between 2014 and 2021.

## 2 METHODS

We conducted the Living Database of HIV Implementation Science (LIVE) project, which was a systematic review of studies evaluating HIV implementation strategies that was repeated at yearly intervals from 2014 to 2021. The project aimed to support the generation of rapid living systematic reviews and characterize the landscape of HIV implementation strategies to identify gaps through systematic searches and data extraction into a living relational database [10]. The data summary from the LIVE systematic review is accessible via a public dashboard (https://live.idig.science/) with the entire dataset of individual study‐level data exportable from the backend upon request. This analysis included all studies that were published between 1 January 2014 and 27 August 2021, and underwent the LIVE systematic review process, with the individual study‐level data exported from the LIVE database on 20 April 2023, for analysis. Below, we reported the methods of the LIVE systematic review and findings according to the Preferred Reporting Items for Systematic Reviews and Meta‐Analyses (PRISMA) reporting guidelines [11] (Supporting information S1).

### 2.1 Search strategy

We searched PubMed, Embase and CINAHL for English language studies using separate search strings for HIV/AIDS, LMICs and HIV outcomes. The full search strategy for all databases is presented in Supporting information S2.

### 2.2 Study selection and screening

Studies were eligible if they met the following criteria: published in the English language, conducted in any LMICs as defined by the World Bank [12], described the implementation of HIV intervention and assessed at least one HIV cascade outcome (i.e. HIV testing, HIV diagnosis, linkage to care, ART initiation, ART adherence, retention in care, re‐engagement in care and viral suppression). The studies could be conducted in any populations and age groups, and using any study designs. Duplicate studies were removed prior to screening. Conference abstracts and studies with unavailable full texts were excluded during full‐text screening.

During the screening process on Covidence (Veritas Health Innovation Ltd, Melbourne, Australia), ≥2 trained researchers screened each abstract and ≥2 trained researchers screened the full text and noted reasons for exclusions. Disagreements were resolved through discussion or by a third reviewer.

### 2.3 Data extraction

Once the screening was completed, ≥1 trained researcher inductively extracted study and strategy characteristics from full texts into a relational database, AirTable (Formagrid Inc., San Francisco, CA). Full‐text publications relating to the same study were treated as one study and their data were extracted together. At the study level, we collected authors, study title, publication source, publication date, enrolment dates, study design, study country(ies), urban‐rural setting(s), study population(s), sample size and intervention arm(s). At the strategy level, we collected actor(s), action, action target(s) [3], location(s), delivery mode(s) [3] and HIV care cascade target(s) (Table 1). We conducted a systematic quality control check of 25% of studies for accuracy and consistency. All strategies from the included studies underwent an independent quality control check by a second member of the research team.

**Table 1:** Description of strategy components extracted in the Living Database of HIV Implementation Science systematic review of HIV implementation strategies

Strategy components	Description
Actor(s)	An individual or a group of stakeholders who deliver the strategy [3]
Action	An activity or process that is being performed [3]
Action target(s)	An individual or a group of stakeholders who are impacted by the strategy [3]
Location(s)	The place where the actors deliver the action
Delivery mode(s)	The approach in which the actors deliver the action (e.g. in‐person, telephone, SMS)
HIV care cascade target(s)	The steps in the HIV care continuum (i.e. HIV testing, HIV diagnosis, linkage to care, antiretroviral therapy initiation, antiretroviral therapy adherence, retention in care, re‐engagement in care, viral suppression)

### 2.4 Analysis

For each intervention arm within a study, multifaceted strategies were separated into discrete strategies, which were defined as a unique combination of individual actor, action and action target. Duplicate strategies within a study arm were removed from the analysis. Inductively coded actors and action targets were categorized based on existing taxonomies, including the 7 P's Framework to Identify Stakeholders in patient‐centred outcomes research and comparative effectiveness research [13], and WHO's classification of health workforce [14]. Inductively coded actions were categorized based on the Effective Practice and Organisation of Care [6] and Expert Recommendations for Implementing Change [7, 8] when possible (Supporting information S3). New terminologies were introduced where gaps in taxonomies existed, particularly health service delivery strategies that targeted individual PLWH [15].

Descriptive analysis was conducted to summarize study characteristics and strategy specifications using frequency and percentage for categorical variables and median and range for continuous variables. Heatmaps were generated to visualize the distribution of strategies by actor and action target. We also explored heterogeneity in strategy distributions by publication years, geographic regions, study populations and HIV cascade targets.

The analysis was conducted using R version 4.1.2 (R Foundation for Statistical Computing, Vienna, Austria).

## 3 RESULTS

**Figure 1:**
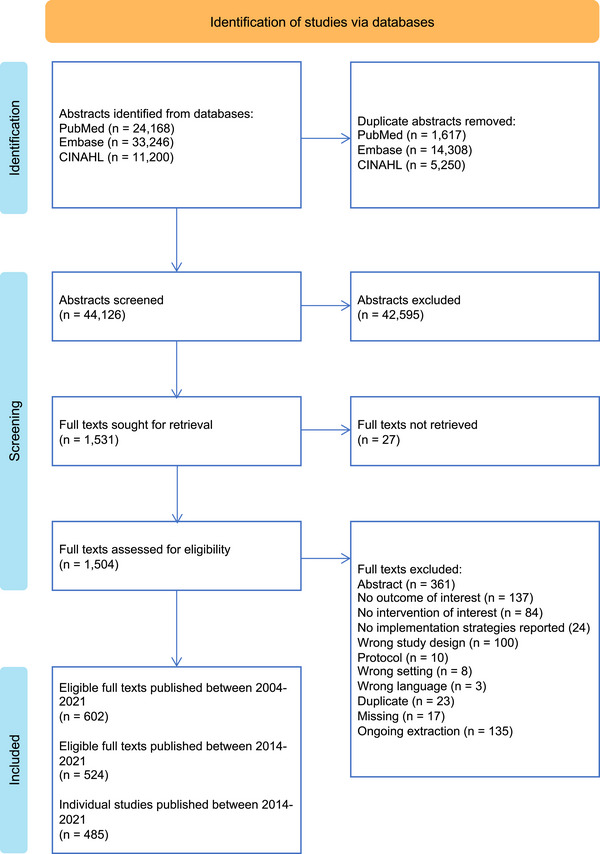
**PRISMA flow chart of the Living Database of HIV Implementation Science systematic review of implementation studies reporting the use of implementation strategies to improve the HIV care cascade outcomes in low‐ and middle‐income countries, 2014–2021**.

After de‐duplication, searches yielded 44,126 records that underwent title and abstract screening. One thousand five hundred and four full texts were assessed for eligibility (Figure 1). A total of 524 eligible full texts published between 2014 and 2021, representing 485 unique studies, were included in this analysis.

**Figure 2:**
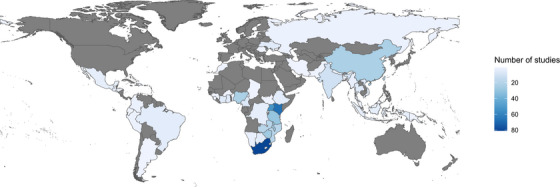
**World map showing low‐ and middle‐income countries where the studies included in the Living Database of HIV Implementation Science systematic review of HIV implementation strategies used to improve the HIV care cascade outcomes, 2014–2021 (*n* = 485)**. The darker shade of blue indicates a higher number of studies, while the lighter shade of blue indicates a lower number of studies.

As shown in Table 2, the most common study designs were randomized controlled trials (44%) and cohort studies (33%). The majority of the studies were conducted in sub‐Saharan Africa (83%; Figure 2). More than half of the studies focused on the general population with few studies targeting key populations, such as pregnant and postpartum women (17%), men who have sex with men (11%) and female sex workers (5%). HIV testing (52%), linkage to care (31%) and retention in care (33%) were the most common HIV cascade targets. From these studies, there were a total of 868 intervention arms with a median number of intervention arms of 2 (range: 1–9) per study. From all intervention arms, 7,253 unique strategies were identified with the median number of strategies per intervention arm of 6 (range: 1–71). A list of studies included in this analysis is presented in Supporting information S4.

**Table 2:** Characteristics of studies from the Living Database of HIV Implementation Science systematic review of HIV implementation strategies used to improve the HIV care cascade outcomes in low‐ and middle‐income countries, 2014–2021 (*N* = 485)

	*n* (%)
**Study design**	
Randomized controlled	211 (44%)
Cohort	159 (33%)
Quasi‐experimental	51 (11%)
Programme evaluation	24 (5%)
Cross‐sectional	22 (5%)
Mixed methods	9 (2%)
Case‐control	7 (1%)
Economic evaluation	2 (0%)
**WHO geographic region**	
Sub‐Saharan African region	404 (83%)
Region of the Americas	39 (8%)
Western Pacific Region	31 (6%)
South‐East Asian Region	28 (6%)
European Region	9 (2%)
Eastern Mediterranean Region	4 (1%)
**Key population**	
No key population	296 (61%)
Pregnant and postpartum women	84 (17%)
Men who have sex with men	52 (11%)
Female sex workers	26 (5%)
People living with HIV and tuberculosis	22 (5%)
People who inject drugs	21 (4%)
Transgender women	15 (3%)
Transgender men	6 (1%)
Migrant/mobile workers	5 (1%)
People with disabilities	5 (1%)
Incarcerated people	2 (0%)
**Age group**	
Adults	412 (85%)
Adolescents/youths	175 (36%)
Children	63 (13%)
Infants	66 (14%)
**HIV cascade target**	
HIV testing	257 (52%)
HIV diagnosis	114 (24%)
Linkage to care	152 (31%)
Antiretroviral therapy initiation	139 (28%)
Antiretroviral therapy adherence	118 (24%)
Retention in care	160 (33%)
Re‐engagement in care	7 (1%)
Viral suppression	75 (15%)
**Publication year**	
2014	47 (10%)
2015	33 (7%)
2016	55 (11%)
2017	66 (14%)
2018	70 (14%)
2019	69 (14%)
2020	78 (16%)
2021	67 (14%)

**Figure 3:**
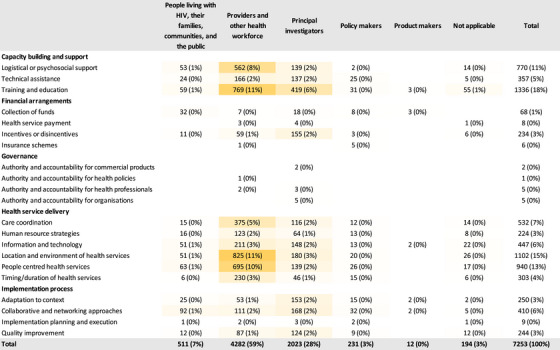
**Distribution of implementation strategies reported in the implementation studies for improved HIV care cascade outcomes, published between 2014 and 2021, and included in the Living Database of HIV Implementation Science systematic review, categorized by existing taxonomy stratified by actors (*N* = 7,253)**. The darker shade of orange indicates a higher proportion of strategies used, while the white colour indicates a low proportion or no strategies used.

**Figure 4:**
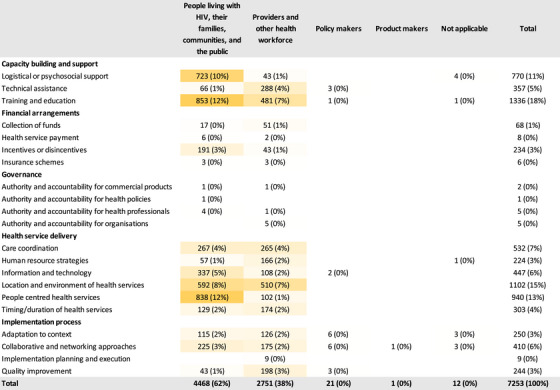
**Distribution of implementation strategies reported in the implementation studies for improved HIV care cascade outcomes, published between 2014 and 2021, and included in the Living Database of HIV Implementation Science systematic review, categorized by existing taxonomy stratified by action targets (*N* = 7,253)**. The darker shade of orange indicates a higher proportion of strategies used, while the white colour indicates a low proportion or no strategies used.

The strategies were grouped into five high‐level categories: capacity building and support (34%), financial arrangements (4%), governance (<1%), health service delivery (48%) and implementation process (12%). Among the five high‐level categories of stakeholders identified to have led implementation strategies, providers and other health workforce (59%), and principal investigators and researchers (28%) enacted the majority of the strategies (Figure 3). Only four broad types of stakeholders served as action targets with PLWH, their families, communities and the public (62%), and providers and other health workforce (38%) being the most common targets (Figure 4). For health service delivery strategies, which accounted for almost half of the strategies identified, providers primarily changed the location and environment of health services (11%), provided people‐centred health services (10%) and coordinated care between providers (5%). Providers themselves and PLWH and their communities were the main targets of these health service delivery strategies. Similarly, for capacity building and support strategies, providers were the main actors and provided training and education (11%), and logistical or psychosocial support (8%), while PLWH and their communities were the targets of these strategies (12% and 10%, respectively). Principal investigators enacted 6% of training and education strategies. Providers also received training and education (7%) as well as technical assistance (4%). The few implementation process strategies reported were enacted primarily by principal investigators in order to adapt intervention to context (2%), use collaborative and networking approaches (2%) and conduct quality improvement (2%). These implementation process strategies targeted providers and PLWH and their communities.

Similar to the characteristics of the studies included in this review, most of the strategies were used in sub‐Saharan Africa (79%; 5,717/7,253), with fewer used in South‐East Asia and Western Pacific (12%; 844/7,253), or the Americas (7%; 514/7,253). Most of the strategies targeted the general populations (60%; 4,334/7,253), while pregnant and postpartum women (17%; 1,251/7,253), men who have sex with men (*n* = 887; 12%; 887/7,253), female sex workers (7%; 489/7,253) and other key populations were less targeted. The number of strategies used decreased with age: adults (86%; 6,254/7,253), adolescents and youths (36%; 2,577/7,253), children (11%; 820/7,253) and infants (12%; 906/7,253). The number of strategies also decreased across the cascade: testing (57%; 4,137/7,253), linkage to care (39%; 2,839/7,253), ART initiation (34%; 2,482/7,253), retention in care (30%; 2,168/7,253), ART adherence (21%; 1,545/7,253) and viral suppression (17%; 1,254/7,253).

The overall strategy distributions were largely consistent over time, geographic regions, study populations and HIV cascade targets with some notable differences. Higher proportion of capacity building and support strategies (47%) and lower proportion of health service delivery strategies (39%) were found among studies targeting ART adherence when compared to the overall proportions of 34% and 48%, respectively. Additionally, higher proportions of implementation process strategies were reported among studies conducted on men who have sex with men (16%) and female sex workers (20%), and a lower proportion in studies conducted among children (8%), when compared to the overall proportion of 12%. There were higher proportions of strategies enacted by PLWH and their communities, and by principal investigators and researchers in studies conducted among men who have sex with men (11% and 36%) and female sex workers (15% and 35%), when compared to the overall proportions of 7% and 28%, respectively. Providers enacted 66–67% of the strategies that targeted ART adherence, retention and viral suppression, which were higher than the overall proportion of 59%. Although policymakers only enacted 3% of the strategies overall, higher proportions of strategies enacted by policymakers were found in studies conducted among infants (9%) and children (7%). In terms of strategy distributions by action targets, there were lower proportions of strategies targeting PLWH and their communities and higher proportions of strategies targeting providers in studies conducted in the Americas (56% and 44%, respectively), among infants (43% and 57%, respectively) and children (51% and 49%, respectively), and targeting ART initiation (56% and 43%, respectively), when compared to the overall proportions of 62% and 38%, respectively.

Implementation strategies in the literature were likely underreported. The number of strategies reported per intervention arm varied from one strategy to 71 strategies. While our research team captured all strategies reported in the full texts, we had difficulty specifying strategies when one activity or process might be considered multiple strategies and when actors and action targets were not explicitly stated, such as when the authors used “we” to represent the actor and used passive voice. These issues required extensive discussions to ensure all strategies and their components were captured as presented in the full texts. Additionally, certain types of implementation strategies might be more underreported than others based on our observation of a skewed distribution of implementation strategies.

## 4 DISCUSSION

Our comprehensive living systematic review of HIV implementation research in LMICs from 2014 to 2021 identified over 7000 instances in which implementation strategies of unique actor‐action‐target combinations from 129 identified unique strategies have been used to improve the HIV care cascade outcomes. The most common strategies attempted were to change health service delivery or provide capacity building and support, which were proximal to the HIV cascade outcomes. Strategies attempting to change governance, financial arrangements and implementation processes were rarely reported. Providers and other health workforce enacted most of the strategies, followed by principal investigators and researchers. PLWH, their families, communities and the public enacted few strategies despite being the primary action targets of the reported strategies. The other primary action targets were providers and other health workforce. Moreover, policymakers and product makers were negligibly involved in published HIV‐related implementation research in LMICs. Taken together, these data suggest that there is a need to evaluate a variety of strategies enacted by and targeted diverse individuals for implementation effectiveness in LMICs with likely relevance to enhance innovation globally.

Most of the identified strategies directly target health service delivery—how an intervention is delivered to PLWH—rather than change the implementation process or environment. Most of the identified health service delivery strategies follow a differentiated service delivery approach in which the type, location, provider and timing of HIV services are tailored to provide various people‐centred HIV service models to meet the diverse needs and/or preferences of PLWH [16, 17, 18]. Another common type of reported strategy, capacity building and support, may be essential in educating PLWH and their communities on how to access and adhere to HIV interventions, and in equipping healthcare providers, including lower cadre and lay providers, with the necessary knowledge and skills to provide the HIV interventions. However, the skewed distribution of implementation strategies suggested that the strategies that change the actual implementation process, financial arrangement and governance [6] that underly how the healthcare organization and health system operate were rarely, if at all, reported in the published studies. Possibly, these strategies might have been performed but were underreported in study manuscripts, or the studies reporting these strategies may have been less likely to meet our eligibility criteria. Nonetheless, findings on strategies that are more distal to the HIV cascade outcomes might provide crucial insights on how the implementation of an HIV intervention could lead to favourable health outcomes. Additionally, these findings may help other implementers understand and adapt the study findings to their own implementation environment. Future studies should incorporate and report all implementation strategies used throughout the pre‐ to post‐implementation phases. Proctor et al. provided a clear framework for strategy specification [3]. Various tools exist to facilitate strategy tracking and documentation over time, which can be done prospectively [19, 20] and retrospectively [21].

The people implementing these published strategies were largely restricted to healthcare providers and researchers as actors, while PLWH and their communities were most commonly the action targets. Other influential stakeholders—including public health and regulatory agencies, the pharmaceutical industry and the media—were rarely reported but should be included purposefully and early on in the research process [22] and implementation efforts in order to ensure that the intervention is acceptable, feasible and sustainable. Greater involvement of PLWH as actors is critical, and has been recommended since 1983 [23]. There remains a need to move beyond treating PLWH as the end users of HIV interventions and position them as the main actors to co‐create, lead, and ultimately be the experts and decision‐makers in the implementation efforts [24]. The key population‐led health service model in Asia has shown how healthcare providers, researchers and policymakers can support the key populations living with HIV and their communities to lead needs‐based, demand‐driven and client‐centred HIV services through capacity building and sustainable financing [25, 26, 27]. This type of approach that engages and empowers affected communities is required globally. In rural Ethiopia, inclusive and active involvement of mental health service users, caregivers and other stakeholders with diverse perspectives and expertise in a participatory action research approach has proved to enhance research participation to strengthen mental health systems [28]. This approach was carried out through the formation of stakeholder groups, capacity building trainings and interactive consultation meetings [28], and could be applied to HIV service users as well. Collectively, this finding calls for meaningful engagement and leadership from a broader spectrum of actors and action targets in HIV implementation research and practice.

The majority of the studies came from sub‐Saharan Africa, South‐East Asia and Western Pacific, which are the regions with high HIV burden that have seen a decrease in the number of HIV acquisitions overall [1]. Few published studies were conducted in Eastern Europe, Central Asia, Middle East and North Africa where the number of HIV acquisitions has been rising in the past decade [1]. This trend potentially demonstrates how implementation strategies could help curb the epidemic or might reflect the outcomes of the investment and funding allocation, which drive the implementation of HIV programmes, in certain regions [29]. Understanding implementation strategy opportunities and lessons from varied geographies is important to equitably address HIV in all geographic areas with different epidemic sizes, dynamics and priorities.

Few studies from our systematic review sought to improve HIV care specifically for key populations despite having a heightened HIV burden globally. Among studies focused on men who have sex with men and female sex workers, higher proportions of PLWH and their communities served as implementation actors compared to the rest of the studies in our sample. Men who have sex with men community leadership and participation have been integral in the advocacy and fought for their rights, health needs and capacity to lead the HIV response since the beginning of the HIV epidemic [30]. Involving and empowering the community to lead the implementation process can inform appropriate service delivery and maintain credibility with key populations [30]. However, few implementation studies thus far have evaluated the implementation strategies to improve the HIV care cascade outcomes of people who inject drugs, transgender people and incarcerated people, who also bear large HIV burdens. Young people and children also have been left behind in the global HIV implementation efforts to end the epidemic with the UNAIDS’ targets lagging behind those of adults [1]. Further HIV programmes should focus on and follow the lead of populations with the greatest HIV burden.

Most of the studies targeted HIV testing, and the number of strategies reported to improve HIV treatment outcomes decreased along the care cascade [31]. As about a third of PLWH in LMICs are lost to care within 3 years, and care attrition increases over time [32], more effective strategies are needed to link people diagnosed with HIV to care [33], initiate ART [33, 34, 35] and ensure ART adherence [31, 36, 37], retention in care and opportunities to re‐engage in care [38] in order to facilitate and sustain viral suppression. Additionally, there were higher proportions of strategies enacted by healthcare providers that targeted the later part of the HIV treatment cascade. Growing evidence supports that capacity building of and task shifting HIV service provision to lower cadre providers, lay providers, community members and PLWH can improve HIV outcomes throughout the care cascade [27, 39, 40, 41, 42]. While the LIVE project focused on strategies that improve HIV treatment outcomes, future research should study HIV prevention strategies as well.

This manuscript was strengthened by the large‐scale and comprehensive living systematic review, which provides a platform for rapid knowledge translation to answer specific research questions related to HIV service delivery in the form of several systematic review and meta‐analysis products, and more importantly to inform cycles of guideline development [10]. However, maintaining a comprehensive living systematic review has been a laborious task given the requirement to annually update the review and maintain the relevance of the project while the research and practice priorities continue to evolve. The latest round of the LIVE systematic review included studies published up until 27 August 2021. We found that the strategy distribution was consistent over time from 2014 to 2021. While the body of literature included in this review reflected lessons learned on HIV treatment service delivery from the pre‐COVID‐19 pandemic period, we believe there has been no substantial change in the use of implementation strategies, although there could possibly be a slight increase in the use of information and technology, such as novel testing and mHealth strategies, and adaptation to context in response to the COVID‐19 pandemic.

There were also other limitations. Firstly, this study was subject to publication bias as not all implementation efforts result in a research publication. Our study also only included studies published in the English language, which is a barrier for knowledge dissemination, especially in non‐English speaking LMICs. Secondly, potential bias from only including full texts reporting HIV cascade outcomes might cause an inflation in the number of strategies targeting PLWH, rather than healthcare providers, policymakers and other action targets. Thirdly, incomplete reporting strategy usage and specification limited us from conducting a formal meta‐analysis to describe the distribution of strategy usage given the possibility of skewed reporting. Fourthly, actor‐action‐action‐target combinations were treated as independent strategies in this study; however, strategies sometimes required multiple actors, actions and action targets. Further research is underway to understand the interplay between strategies and multifaceted strategies. Lastly, we did not assess the effectiveness of the strategies or the interventions implemented in improving the HIV cascade outcomes and this could be an area for further study.

## 5 CONCLUSIONS

Healthcare providers, researchers, PLWH and their communities have been involved in HIV implementation strategies in LMIC. However, many potentially effective strategies have not been reported in the literature. We may consider expanding the strategy usage to ensure that all stakeholders are meaningfully involved throughout the implementation phases and that there are equitable efforts to improve HIV outcomes across the geographic regions, target populations and HIV treatment and prevention cascade. Implementation researchers should support key stakeholders in systematically documenting, reporting and evaluating strategies for dissemination of lessons learned and best practices in order to better inform how we should improve our HIV responses.

## COMPETING INTERESTS

All authors declare no competing interests related to this work.

## AUTHORS’ CONTRIBUTIONS

IE‐W, LB, SS, SB, EHG and CGK led the design and implementation of the LIVE project. IE‐W, NLT, RT and BF developed and maintained the database. NLT and RT led the systematic review process. SL, NLT, RT and AU were part of a team of research assistants who reviewed studies for inclusion and abstracted the data. SL and CGK conducted the analysis. SL wrote the first draft. All authors contributed to the review and revision of the manuscript, and approved the final manuscript.

## FUNDING

This work was supported in part by the Bill and Melinda Gates Foundation (INV‐009840_2020). Under the grant conditions of the Foundation, a Creative Commons Attribution 4.0 Generic License has already been assigned to the Author Accepted Manuscript version that might arise from this submission. This work was also supported bythe Johns Hopkins University Center for AIDS Research (P30AI094189).

## CME STATEMENT

This article is published as part of a supplement supported by unrestricted educational grant by ViiV Healthcare.

Credits Available for this Activity: American Medical Association (AMA Credit).

Washington University School of Medicine in St. Louis designates this enduring material for a maximum of 1 AMA PRA Category 1 Credit™. Physicians should claim only the credit commensurate with the extent of their participation in the activity.

## Supporting information


**Supporting information 1**. PRISMA 2020 Main Checklist

**Supporting information 2**. Search terms for the Living Database of HIV Implementation Science (LIVE)

**Supporting information 3**. Implementation strategies identified in the Living Database of HIV Implementation Science (LIVE) systematic review

**Supporting information 4**. Main and related studies included in the Living Database of HIV Implementation Science (LIVE) systematic review

## Data Availability

The data summary is available at https://idig.science/LIVE/. Individual study‐level data are available upon reasonable request.
