# Supplementary material for: Implementation strategies to improve HIV care cascade outcomes in low‐ and middle‐income countries: a systematic review from 2014 to 2021
Source: J Int AIDS Soc. 2024 Jul 5;27(Suppl 1):e26263. doi: 10.1002/jia2.26263 (PMC11224579; doi:10.1002/jia2.26263)
Supplement: Supplementary file 2 — Supporting information 2. Search terms for the Living Database of HIV Implementation Science (LIVE) [file JIA2-27-e26263-s004.docx]

Appendix 2. Search terms for the Living Database of HIV Implementation Science (LIVE) systematic review of HIV implementation strategies used to improve the HIV care cascade outcomes in low- and middle-income countries, 2004-2021.

Database – Embase: Searched 08/27/2021

Limiters: Publication – 01/01/2004-08/27/2021; English only, Humans

| Action | Term |
| --- | --- |
| 1 | ('human immunodeficiency virus infection'/exp OR 'human immunodeficiency virus infection' OR 'acquired immune deficiency syndrome'/exp OR 'acquired immune deficiency syndrome' OR 'human immunodeficiency virus 1'/exp OR 'human immunodeficiency virus 1' OR 'human immunodeficiency virus 2'/exp OR 'human immunodeficiency virus 2' OR 'human immunodeficiency virus'/exp OR 'human immunodeficiency virus') AND limiters |
| 2 | ('hiv-1*':ti,ab OR 'hiv-2*':ti,ab OR 'hiv':ti,ab OR 'hiv/aids':ti,ab) AND limiters |
| 3 | ('human immunodeficiency virus':ti,ab OR 'human immune deficiency virus':ti,ab OR 'human immuno-deficiency virus':ti,ab OR 'human immune-deficiency virus':ti,ab) AND limiters |
| 4 | ('acquired immune deficiency syndrome':ti,ab OR 'acquired immunodeficiency syndrome':ti,ab OR 'acquired immune-deficiency syndrome':ti,ab OR 'acquired immuno-deficiency syndrome':ti,ab) AND limiters |
| 5 | Or/1-4 |
| 6 | ('diagnostic test'/exp OR 'self evaluation'/exp OR 'diagnostic test*':ti,ab OR 'self eval*':ti,ab OR 'hiv test*':ti,ab) AND limiters |
| 7 | ('preventive medicine'/exp OR 'preventive medicine':ti,ab OR 'preventive health':ti,ab) AND limiters |
| 8 | ('fatality'/exp OR 'mortality'/exp OR 'fatal outcome':ti,ab OR 'mortality outcome':ti,ab OR 'treatment outcome'/exp OR 'treatment outcome':ti,ab) AND limiters |
| 9 | ('prognosis'/exp/mj OR 'early diagnosis'/exp/mj OR 'outcome assessment'/exp/mj) AND limiters |
| 10 | ('retention in care'/exp OR 'retention in care':ti,ab OR 'engagement in care':ti,ab) AND limiters |
| 11 | ('time to treatment'/exp OR 'patient care'/exp/mj OR 'treatment initiation':ti,ab OR 'continuum of care':ti,ab) AND limiters |
| 12 | ('loss to follow up'/exp OR 'loss to care':ti,ab OR 'loss to program':ti,ab OR 'ltfu':ti,ab) AND limiters |
| 13 | ('link to care':ti,ab OR 'link to treatment':ti,ab OR 'linkage to treatment':ti,ab OR 'link into treatment':ti,ab) AND limiters |
| 14 | ('patient compliance'/exp OR 'adherence to medication':ti,ab OR 'treatment adherence':ti,ab OR 'patient compliance':ti,ab) AND limiters |
| 15 | ('attitude to health'/exp OR 'sustained virologic response'/exp OR 'antiretroviral therapy'/exp OR 'art drug therapy':ti,ab OR 'antiretroviral treatment':ti,ab) AND limiters |
| 16 | ('implementation science'/exp OR 'implementation science':ti,ab OR 'implementation program':ti,ab OR 'implementation research':ti,ab OR 'implementation strateg*':ti,ab) AND limiters |
| 17 | Or/ 6-16 |
| 18 | ('resource allocation'/exp OR 'developing country'/exp OR 'low middle income country'/exp OR 'poverty'/exp) AND limiters |
| 19 | ('lmic':ti,ab OR 'low middle income countr*':ti,ab) AND limiters |
| 20 | ('developing countr*':ti,ab OR 'least developed countr*':ti,ab OR 'less developed countr*':ti,ab OR 'under developed countr*':ti,ab OR 'third world countr*':ti,ab) AND limiters |
| 21 | ('developing nation*':ti,ab OR 'least developed nation*':ti,ab OR 'less developed nation*':ti,ab OR 'under developed nation*':ti,ab OR 'third world nation*':ti,ab) AND limiters |
| 22 | ('afghanistan':ti,ab OR 'albania':ti,ab OR 'algeria':ti,ab OR 'american somoa':ti,ab OR 'angola':ti,ab OR 'argentina':ti,ab OR 'armenia':ti,ab OR 'azerbaijan':ti,ab OR 'bangladesh':ti,ab OR 'belarus':ti,ab OR 'belize':ti,ab OR 'benin':ti,ab OR 'bhutan':ti,ab OR 'bolivia':ti,ab OR 'bosnia herzegovina':ti,ab OR 'botswana':ti,ab OR 'brazil':ti,ab OR 'bulgaria':ti,ab OR 'burkina faso':ti,ab OR 'burundi':ti,ab OR 'cabo verde':ti,ab OR 'cambodia':ti,ab OR 'cameroon':ti,ab OR 'central african republic':ti,ab OR 'chad':ti,ab OR 'china':ti,ab OR 'colombia':ti,ab OR 'comoros':ti,ab OR 'republic of congo':ti,ab OR 'costa rica':ti,ab OR 'cote divoire':ti,ab OR 'cuba':ti,ab) AND limiters |
| 23 | ('djibouti':ti,ab OR 'dominica':ti,ab OR 'dominican republic':ti,ab OR 'ecuador':ti,ab OR 'egypt':ti,ab OR 'el salvador':ti,ab OR 'equatorial guinea':ti,ab OR 'eritrea':ti,ab OR 'eswatini':ti,ab OR 'ethiopia':ti,ab OR 'fiji':ti,ab OR 'gambia':ti,ab OR 'georgia':ti,ab OR 'ghana':ti,ab OR 'grenada':ti,ab OR 'guatemala':ti,ab OR 'guinea':ti,ab OR 'guinea-bissau':ti,ab OR 'guyana':ti,ab OR 'haiti':ti,ab OR 'honduras':ti,ab OR 'india':ti,ab OR 'indonesia':ti,ab OR 'iran':ti,ab OR 'iraq':ti,ab OR 'jamaica':ti,ab OR 'jordan':ti,ab OR 'kazakhastan':ti,ab OR 'kenya':ti,ab OR 'kiribati':ti,ab OR 'north korea':ti,ab OR 'kosovo':ti,ab OR 'kyrgyz':ti,ab) AND limiters |
| 24 | ('laos':ti,ab OR 'lebanon':ti,ab OR 'lesotho':ti,ab OR 'liberia':ti,ab OR 'libya':ti,ab OR 'madagascar':ti,ab OR 'malawi':ti,ab OR 'malaysia':ti,ab OR 'maldives':ti,ab OR 'mali':ti,ab OR 'marshall islands':ti,ab OR 'mauritania':ti,ab OR 'mauritius':ti,ab OR 'mexico':ti,ab OR 'micronesia':ti,ab OR 'moldova':ti,ab OR 'mongolia':ti,ab OR 'montenegro':ti,ab OR 'morocco':ti,ab OR 'mozambique':ti,ab OR 'myanmar':ti,ab OR 'namibia':ti,ab OR 'nauru':ti,ab OR 'nepal':ti,ab OR 'nicaragua':ti,ab OR 'niger':ti,ab OR 'nigeria':ti,ab OR 'north macedonia':ti,ab OR 'pakistan':ti,ab OR 'papua new guinea':ti,ab OR 'paraguay':ti,ab OR 'peru':ti,ab OR 'philippines':ti,ab OR 'romania':ti,ab OR 'russia':ti,ab OR 'rwanda':ti,ab) AND limiters |
| 25 | ('samoa':ti,ab OR 'sao tome':ti,ab OR 'senegal':ti,ab OR 'serbia':ti,ab OR 'sierra leone':ti,ab OR 'solomon islands':ti,ab OR 'somalia':ti,ab OR 'south africa':ti,ab OR 'south sudan':ti,ab OR 'sri lanka':ti,ab OR 'st lucia':ti,ab OR 'st vincent':ti,ab OR 'sudan':ti,ab OR 'suriname':ti,ab OR 'syria':ti,ab OR 'tajikistan':ti,ab OR 'tanzania':ti,ab OR 'thailand':ti,ab OR 'timor-leste':ti,ab OR 'togo':ti,ab OR 'tonga':ti,ab OR 'tunisia':ti,ab OR 'turkey':ti,ab OR 'turkmenistan':ti,ab OR 'tuvalu':ti,ab OR 'uganda':ti,ab OR 'ukraine':ti,ab OR 'uzbekistan':ti,ab OR 'vanuatu':ti,ab OR 'venezuela' OR 'vietnam':ti,ab OR 'west bank gaza':ti,ab OR 'yemen':ti,ab OR 'zambia':ti,ab OR zimbabwe:ti,ab) AND limiters |
| 26 | OR/18-25 |
| 27 | 5 AND 17 AND 26 |

Database – PubMed: Searched 08/27/2021

Limiters: Publication – 01/01/2004-08/27/2021; English only, Humans

| Action | Term |
| --- | --- |
| 1 | (“Hiv” [Mesh] OR “HIV Infections” [Mesh] OR “acquired immunodeficiency syndrome” [Mesh]) with limiter |
| 2 | ("hiv-1"[Title/Abstract] OR "hiv-2"[Title/Abstract] OR "hiv"[Title/Abstract] OR "hiv aids"[Title/Abstract]) OR ("hiv-1"[Text Word] OR "hiv-2"[Text Word] OR "hiv"[Text Word] OR "hiv aids"[Text Word]) with limiter |
| 3 | ("hiv infec*"[Title/Abstract] OR "AIDS virus"[Title/Abstract] OR CD4[Title/Abstract]) OR ("hiv infec*"[Text Word] OR "AIDS virus"[Text Word] OR CD4[Text Word]) with limiter |
| 4 | ("human immunodeficiency virus"[Title/Abstract] OR "human immune deficiency virus"[Title/Abstract] OR "human immuno-deficiency virus"[Title/Abstract] OR "human immune-deficiency virus"[Title/Abstract]) OR ("human immunodeficiency virus"[Text Word] OR "human immune deficiency virus"[Text Word] OR "human immuno-deficiency virus"[Text Word] OR "human immune-deficiency virus"[Text Word]) with limiters |
| 5 | ("acquired immunodeficiency syndrome"[Title/Abstract] OR "acquired immune deficiency syndrome"[Title/Abstract] OR "acquired immune-deficiency syndrome"[Title/Abstract] OR "acquired immune-deficiency syndrome"[Title/Abstract]) OR ("acquired immunodeficiency syndrome"[Text Word] OR "acquired immune deficiency syndrome"[Text Word] OR "acquired immune-deficiency syndrome"[Text Word] OR "acquired immune-deficiency syndrome"[Text Word]) with limiters |
| 6 | Or/1-5 |
| 7 | "diagnostic tests, routine"[MeSH Terms] OR "diagnostic test*"[Title/Abstract] OR "HIV test*"[Title/Abstract] OR "self-testing"[Title/Abstract] with limiters |
| 8 | "Preventive Medicine"[MeSH Terms] OR "preventive medicine"[Title/Abstract] with limiters |
| 9 | "Fatal Outcome"[MeSH Terms] OR "mortality"[Title/Abstract] OR "fatal outcome"[Title/Abstract] with limiters |
| 10 | Treatment Outcome"[MeSH Terms] OR "treatment outcome"[Title/Abstract] with limiters |
| 11 | (("prognosis"[MeSH Terms]) OR ("Early diagnosis"[MeSH Major Topic])) OR ("outcome assessment, health care"[MeSH Terms]) with limiters |
| 12 | "Retention in Care” [MeSH Terms] OR "retention in care"[Title/Abstract] OR "engagement in care"[Title/Abstract] with limiters |
| 13 | "Time-to-Treatment"[MeSH Terms] or "treatment initiation"[Title/Abstract] OR "continuum of care" [Title/Abstract] with limiters |
| 14 | "loss to care"[Title/Abstract] OR "loss to follow up"[Title/Abstract] OR "loss to program"[Title/Abstract] OR "lost to follow-up"[Title/Abstract] OR "LTFU"[Title/Abstract] with limiters |
| 15 | "link to care"[Title/Abstract] OR "link to treatment"[Title/Abstract] OR "linkage to treatment"[Title/Abstract] OR "link into treatment"[Title/Abstract] with limiters |
| 16 | "treatment adherence and compliance"[MeSH Terms] OR "treatment adherence"[Title/Abstract] OR "adherence to medication"[Title/Abstract] with limiters |
| 17 | ("attitude to health"[MeSH Terms]) or ("Sustained Virologic Response"[MeSH Major Topic]) with limiters |
| 18 | "Antiretroviral Therapy, Highly Active"[MeSH Terms] OR "antiretroviral therapy"[Title/Abstract] OR "antiretroviral treatment"[Title/Abstract] with limiters |
| 19 | "Implementation science"[MeSH Terms] OR "implementation science"[Title/Abstract] OR "Implementation research"[Title/Abstract] OR "implementation program"[Title/Abstract] OR "implementation strateg*"[Title/Abstract] with limiters |
| 20 | Or/ 7-19 |
| 21 | "Resource allocation"[MeSH Terms] OR "developing countries"[MeSH Terms] or "poverty"[MeSH Terms] or "poverty areas"[MeSH Terms] with limiters |
| 22 | (LMIC[Title/Abstract] OR “low middle income countr*”[Title/Abstract]) OR (LMIC[Text Word] OR "low middle income countr*"[Text Word]) with limiters |
| 23 | ("Developing countr*"[Title/Abstract] OR "least developed countr*"[Title/Abstract] OR "less developed countr*"[Title/Abstract] OR "under developed countr*"[Title/Abstract] OR "third world countr*"[Title/Abstract]) OR ("Developing countr*"[Text Word] OR "least developed countr*"[Text Word] OR "less developed countr*"[Text Word] OR "under developed countr*"[Text Word] OR "third world countr*"[Text Word]) with limiters |
| 24 | ("Developing nation*"[Title/Abstract] OR "least developed nation*"[Title/Abstract] OR "less developed nation*"[Title/Abstract] OR "under developed nation*"[Title/Abstract] OR "third world nation"[Title/Abstract]) OR ("Developing nation*"[Text Word] OR "least developed nation*"[Text Word] OR "less developed nation*"[Text Word] OR "under developed nation*"[Text Word] OR "third world nation"[Text Word]) with limiters |
| 25 | Afghanistan[Title/Abstract] OR Albania[Title/Abstract] OR algeria[Title/Abstract] OR "American Somoa"[Title/Abstract] OR Angola[Title/Abstract] OR argentina[Title/Abstract] OR armenia[Title/Abstract] OR azerbaijan[Title/Abstract] OR Bangladesh[Title/Abstract] OR Belarus[Title/Abstract] OR belize[Title/Abstract] OR Benin[Title/Abstract] OR Bhutan[Title/Abstract] OR Bolivia[Title/Abstract] OR "Bosnia Herzegovina"[Title/Abstract] OR botswana[Title/Abstract] OR brazil[Title/Abstract] OR Bulgaria[Title/Abstract] OR "Burkina Faso"[Title/Abstract] OR Burundi[Title/Abstract] OR "Cabo Verde"[Title/Abstract] OR Cambodia[Title/Abstract] OR Cameroon[Title/Abstract] OR "Central African Republic"[Title/Abstract] OR Chad[Title/Abstract] OR china[Title/Abstract] OR Colombia OR Comoros[Title/Abstract] OR "Republic of Congo"[Title/Abstract] OR costa rica[Title/Abstract] OR "Cote d Ivoire"[Title/Abstract] OR cuba[Title/Abstract] with limiters |
| 26 | Djibouti[Title/Abstract] OR dominica[Title/Abstract] OR "dominican republic"[Title/Abstract] OR Ecuador[Title/Abstract] OR Egypt[Title/Abstract] OR "El Salvador"[Title/Abstract] OR "equatorial guinea"[Title/Abstract] OR Eritrea[Title/Abstract] OR Eswatini[Title/Abstract] OR Ethiopia[Title/Abstract] OR Fiji[Title/Abstract] OR gabon[Title/Abstract] OR Gambia[Title/Abstract] OR Georgia[Title/Abstract] OR Ghana[Title/Abstract] OR grenada[Title/Abstract] OR Guatemala[Title/Abstract] OR Guinea[Title/Abstract] OR "Guniea-Bissau"[Title/Abstract] OR Guyana[Title/Abstract] OR haiti[Title/Abstract] OR Honduras[Title/Abstract] OR india[Title/Abstract] OR indonesia[Title/Abstract] OR iran[Title/Abstract] OR Iraq[Title/Abstract] OR Jamaica[Title/Abstract] OR Jordan[Title/Abstract] OR kazakhastan[Title/Abstract] OR kenya[Title/Abstract] OR Kiribati[Title/Abstract] OR "North Korea"[Title/Abstract] OR Kosovo[Title/Abstract] OR Kyrgyz[Title/Abstract] with limiters |
| 27 | laos[Title/Abstract] OR Lebanon[Title/Abstract] OR lesotho[Title/Abstract] OR liberia[Title/Abstract] OR libya[Title/Abstract] OR madagascar[Title/Abstract] OR Malawi[Title/Abstract] OR Malaysia[Title/Abstract] OR Maldives[Title/Abstract] OR mali[Title/Abstract] OR "marshall islands"[Title/Abstract] OR Mauritania[Title/Abstract] OR Mauritius[Title/Abstract] OR mexico[Title/Abstract] OR micronesia[Title/Abstract] OR moldova[Title/Abstract] OR mongolia[Title/Abstract] OR Montenegro[Title/Abstract] OR morocco[Title/Abstract] OR Mozambique[Title/Abstract] OR myanmar[Title/Abstract] OR namibia[Title/Abstract] OR Nauru[Title/Abstract] OR nepal[Title/Abstract] OR nicaragua[Title/Abstract] OR niger[Title/Abstract] OR nigeria[Title/Abstract] OR "north Macedonia"[Title/Abstract] OR pakistan[Title/Abstract] OR "papua new guinea"[Title/Abstract] OR Paraguay[Title/Abstract] OR peru[Title/Abstract] OR philippines[Title/Abstract] OR Romania[Title/Abstract] OR russia[Title/Abstract] OR rwanda[Title/Abstract] with limiters |
| 28 | samoa[Title/Abstract] OR "Sao tome"[Title/Abstract] OR senegal[Title/Abstract] OR Serbia[Title/Abstract] OR "sierra leone"[Title/Abstract] OR "solomon islands"[Title/Abstract] OR somalia[Title/Abstract] OR "south Africa"[Title/Abstract] OR "south sudan"[Title/Abstract] OR "sri lanka"[Title/Abstract] OR "st lucia"[Title/Abstract] OR "st Vincent"[Title/Abstract] OR sudan[Title/Abstract] OR suriname[Title/Abstract] OR syria[Title/Abstract] OR tajikistan[Title/Abstract] OR tanzania[Title/Abstract] OR Thailand[Title/Abstract] OR "timor-leste"[Title/Abstract] OR togo[Title/Abstract] OR tonga[Title/Abstract] OR tunisia[Title/Abstract] OR turkey[Title/Abstract] OR Turkmenistan OR Tuvalu[Title/Abstract] OR uganda[Title/Abstract] OR ukraine[Title/Abstract] OR uzbekistan[Title/Abstract] OR vanuatu[Title/Abstract] OR Venezuela[Title/Abstract] OR vietnam[Title/Abstract] OR "west bank gaza"[Title/Abstract] OR yemen[Title/Abstract] OR zambia[Title/Abstract] OR zimbabwe[Title/Abstract] with limiters |
| 29 | Or/21-28 |
| 30 | 6 AND 20 And 29 |

Database – CINAHL: Searched 08/27/2021

Limiters: Publication – 01/01/2004-08/27/2021; English only, Humans

| Action | Term |
| --- | --- |
| 1 | (MH "Human Immunodeficiency Virus+") OR "human immunodeficiency virus infection" OR (MH "HIV-Infected Patients+") OR (MH "HIV Infections+") OR (MM "Acquired Immunodeficiency Syndrome") OR "acquired immune deficiency syndrome" |
| 2 | "hiv/aids" OR (MM "AIDS Patients") OR "AIDS patients" OR "HIV-1*" OR "HIV-2*" OR "HIV" OR "CD4" OR "hiv infec*" OR "aids virus" |
| 3 | "human immunodeficiency virus" OR "human immune deficiency virus" OR "human immuno-deficiency virus" OR "human immune-deficiency virus" OR "acquired immune deficiency syndrome" OR acquired immunodeficiency syndrome" OR "acquired immune-deficiency syndrome" OR acquired immuno-deficiency syndrome" |
| 4 | Or/1-3 |
| 5 | (MM "Diagnostic Tests, Routine") OR "diagnostic test" OR (MM "Self Assessment") OR (MH "Patient Assessment") OR “HIV test*” |
| 6 | (MH "Preventive Health Care+") OR "preventive medicine" |
| 7 | (MM "Fatal Outcome") OR "fatal outcome" OR (MH "Treatment Outcomes+") OR (MH "Mortality+") |
| 8 | MM "Prognosis") OR "prognosis" OR (MM "Early Diagnosis") |
| 9 | (MM "Outcome Assessment") OR "outcome assessment" OR (MH "Outcomes (Health Care)+") |
| 10 | "retention in care" OR "engagement in care" |
| 11 | "time to treatment" OR (MH "Turnaround Time") OR (MH "Patient Care") OR "patient care" OR (MH "Patient Centered Care") OR (MH "Continuity of Patient Care") OR (MH "Treatment Duration") OR (MH "Treatment Refusal") OR (MH "Treatment Complications, Delayed") OR (MH "Treatment Termination") OR "treatment initiation" |
| 12 | "loss to follow up" OR "loss to care" OR "loss to program" OR "ltfu" |
| 13 | "link to care" OR "link to treatment" OR "linkage to treatment" OR "link into treatment" |
| 14 | (MM "Medication Compliance") OR (MM "Attitude to Medical Treatment") OR "adherence to medication" OR (MH "Patient Compliance+") OR "patient compliance" OR "treatment adherence" |
| 15 | (MH "Attitude to Health+") OR "attitude to health" OR "sustained virologic response" OR (MM "Antiretroviral Therapy, Highly Active") OR "antiretroviral therapy" OR (MH "Anti-HIV Agents+") |
| 16 | (MM "Implementation Science") OR "implementation science" OR "implementation program" OR (MM "Program Implementation") OR "implementation research" OR "implementation strateg*" |
| 17 | OR/5-16 |
| 18 | (MM "Developing Countries") OR (MM "Low and Middle Income Countries") or poverty areas or poverty |
| 19 | "lmic" OR "low middle income countr*" |
| 20 | "developing countr*" OR "least developed countr*" OR "less developed countr*" OR "under developed countr*" OR "third world countr*" |
| 21 | "developing nation*" OR "least developed nation*" OR "less developed nation*" OR "under developed nation*" OR "third world nation*" |
| 22 | afghanistan OR albania OR algeria OR "american samoa" OR angola OR argentina OR armenia OR azerbaijan OR bangladesh OR belarus OR belize OR benin OR bhutan OR bolivia OR "bosnia herzegovina" OR botswana OR brazil OR bulgaria OR "burkina faso" OR burundi OR "cabo verde" OR cambodia OR cameroon OR "central african republic" OR chad OR china OR colombia OR comoros OR "republic of congo" OR "costa rica" OR "cote divoire" OR cuba |
| 23 | djibouti OR dominica OR "dominican republic" OR ecuador OR egypt OR "el salvador" OR "equatorial guinea" OR eritrea OR eswatini OR ethiopia OR fiji OR gambia OR georgia OR ghana OR grenada OR guatemala OR guinea OR "guine-bissau" OR guyana OR haiti OR honduras OR india OR indonesia OR iran OR iraq OR jamaica OR jordan OR kazakhastan OR kenya OR kiribati OR "north korea" OR kosovo OR kyrgyz |
| 24 | laos OR Lebanon OR Lesotho OR Liberia OR Libya OR Madagascar OR Malawi OR Malaysia OR Maldives OR Mali OR “marshall islands” OR Mauritania OR Mauritius OR Mexico OR Micronesia OR Moldova OR Mongolia OR Montenegro OR Morocco OR Mozambique OR Myanmar OR Namibia OR Nauru OR Nepal OR Nicaragua OR Niger OR Nigeria OR “north Macedonia” OR Pakistan OR “Papua New guinea” OR Paraguay OR Peru OR Philippines OR Romania OR Russia OR Rwanda |
| 25 | Samoa OR “Sao Tome” OR Senegal OR Serbia OR “sierra leone” OR “Solomon islands” OR Somalia OR “south Africa” OR “south sudan” OR “sri Lanka” OR “st Lucia” OR “st Vincent” OR sudan OR suriname OR Syria OR Tajikistan OR Tanzania OR Thailand OR “timor-leste” OR togo OR tonga OR Tunisia OR turkey OR Turkmenistan OR Tuvalu OR Uganda OR Ukraine OR Uzbekistan OR Vanuatu OR Venezuela OR Vietnam OR “west bank gaza” OR yemen OR Zambia OR Zimbabwe |
| 26 | Or/18-25 |
| 27 | 4 AND 17 AND 26 |
