# Supplementary material for: Implementation strategies to improve HIV care cascade outcomes in low‐ and middle‐income countries: a systematic review from 2014 to 2021
Source: J Int AIDS Soc. 2024 Jul 5;27(Suppl 1):e26263. doi: 10.1002/jia2.26263 (PMC11224579; doi:10.1002/jia2.26263)
Supplement: Supplementary file 3 — Supporting information 3. Implementation strategies identified in the Living Database of HIV Implementation Science (LIVE) systematic review [file JIA2-27-e26263-s002.docx]

**Appendix 3.** The 129 unique implementation strategies identified in the Living Database of HIV Implementation Science (LIVE) systematic review mapped to existing implementation strategy taxonomies, Effective Practice and Organisation of Care (EPOC) and Expert Recommendations for Implementing Change (ERIC). The strategy mapping was conducted independently by three trained implementation researchers (SL, IE, CK). Any discrepancy in mapping were resolved through discussion. Wherever possible, we revised the terminology of the level 1 LIVE strategies to match the level 1 EPOC or ERIC strategies. Similarly, level 1 LIVE strategies were categorised into LIVE strategy levels 2 and 3 based on EPOC levels 2 and 3, and ERIC level 2, wherever possible.

| **LIVE strategy level 1** | **LIVE strategy level 2** | **LIVE strategy level 3** | **EPOC strategy level 1** | **ERIC strategy level 1** |
| --- | --- | --- | --- | --- |
| Assist with health system navigation | Logistical or psychosocial support | Capacity building and support | Case management |  |
| Identify or assign a treatment supporter | Logistical or psychosocial support | Capacity building and support | Self-management | Prepare patients/consumers to be active participants |
| Monitor adherence - unspecified tool or approach | Logistical or psychosocial support | Capacity building and support |  | Intervene with patients/consumers to enhance uptake and adherence |
| Monitor adherence using diary cards | Logistical or psychosocial support | Capacity building and support |  | Intervene with patients/consumers to enhance uptake and adherence |
| Monitor adherence using direct observation therapy (DOT) | Logistical or psychosocial support | Capacity building and support |  | Intervene with patients/consumers to enhance uptake and adherence |
| Provide psychosocial support counselling | Logistical or psychosocial support | Capacity building and support |  | Intervene with patients/consumers to enhance uptake and adherence |
| Provide Transportation | Logistical or psychosocial support | Capacity building and support | Transportation services |  |
| Send reminders | Logistical or psychosocial support | Capacity building and support | Reminders | Remind clinicians |
| Centralise technical assistance | Technical assistance | Capacity building and support |  | Centralize technical assistance |
| Provide checklists | Technical assistance | Capacity building and support | Reminders | Remind clinicians |
| Provide decision support tool/s | Technical assistance | Capacity building and support | Reminders | Remind clinicians |
| Provide guidance document/toolkit | Technical assistance | Capacity building and support | Clinical Practice Guidelines | Distribute educational materials |
| Provide local technical assistance | Technical assistance | Capacity building and support |  | Provide local technical assistance |
| Provide standard operating procedures | Technical assistance | Capacity building and support | Clinical Practice Guidelines | Develop educational materials |
| Supervise/mentor/coach/facilitate | Technical assistance | Capacity building and support | Managerial supervision | Provide clinical supervision |
| Conduct educational outreach visits | Training and education | Capacity building and support | Educational outreach visits, or academic detailing. | Conduct educational outreach visits |
| Conduct ongoing training | Training and education | Capacity building and support | Educational meetings | Conduct ongoing training |
| Distribute educational materials | Training and education | Capacity building and support | Educational materials | Distribute educational materials |
| Make training dynamic | Training and education | Capacity building and support | Educational games | Make training dynamic |
| Provide education on a health innovation/service/behaviour | Training and education | Capacity building and support | Educational meetings | Conduct educational meetings |
| Provide leadership training | Training and education | Capacity building and support | Educational meetings | Recruit, designate, and train for leadership |
| Provide ongoing consultation | Training and education | Capacity building and support | Educational outreach visits, or academic detailing. | Provide ongoing consultation |
| Provide training on people-centred communication | Training and education | Capacity building and support | Educational meetings | Conduct educational meetings |
| Shadow other experts | Training and education | Capacity building and support |  | Shadow other experts |
| Train on clinical practice guidelines | Training and education | Capacity building and support | Educational meetings | Conduct educational meetings |
| Train the trainer | Training and education | Capacity building and support | Educational meetings | Use train-the-trainer strategies |
| Train to learn a new skill | Training and education | Capacity building and support | Educational meetings | Conduct educational meetings |
| Train to refresh skills | Training and education | Capacity building and support | Educational meetings | Conduct educational meetings |
| Provide/alter community loan funds | Collection of funds | Financial arrangements | Community loan funds | Access new funding |
| Provide/alter external funding | Collection of funds | Financial arrangements | External funding | Access new funding |
| Provide/alter health saving accounts | Collection of funds | Financial arrangements | Health savings accounts | Access new funding |
| Provide/alter prepaid funding | Collection of funds | Financial arrangements | Prepaid funding | Access new funding |
| Provide/alter user fees or out of pocket payments | Collection of funds | Financial arrangements | User fees or out of pocket payments | Access new funding |
| Change payment methods for  health workers | Health service payment | Financial arrangements | Payment methods for health workers |  |
| Provide/alter voucher schemes | Health service payment | Financial arrangements | Voucher schemes |  |
| Adjust fund holding | Incentives or disincentives | Financial arrangements | Fund holding |  |
| Institute disincentives | Incentives or disincentives | Financial arrangements |  | Develop disincentives |
| Provide incentive: cash (unconditional) | Incentives or disincentives | Financial arrangements |  |  |
| Provide incentive: conditional cash transfer | Incentives or disincentives | Financial arrangements | Conditional cash transfers |  |
| Provide incentive: other goods and services (unconditional) | Incentives or disincentives | Financial arrangements | Conditional cash transfers |  |
| Provide incentive: pay for performance | Incentives or disincentives | Financial arrangements | Pay for performance – target payments | Alter incentive/allowance structures |
| Provide incentive: salary increase or bonus | Incentives or disincentives | Financial arrangements | Pay for performance – target payments | Alter incentive/allowance structures |
| Provide community-based health insurance | Insurance schemes | Financial arrangements | Community based health insurance | Use other payment schemes |
| Provide social health insurance | Insurance schemes | Financial arrangements | Social health insurance | Use other payment schemes |
| Change procedures for registration | Authority and accountability for commercial products | Governance | Registration |  |
| Enact policies to manage absenteeism | Authority and accountability for health policies | Governance | Policies to manage absenteeism |  |
| Involve stakeholders in policy decisions | Authority and accountability for health policies | Governance | Stakeholder involvement in policy decisions |  |
| Create or change credentialing and/or licensure standards | Authority and accountability for health professionals | Governance | Training and licensing | Create or change credentialing and/or licensure standards |
| Change accreditation or membership requirements | Authority and accountability for organizations | Governance | Accreditation | Change accreditation or membership requirements |
| Regulate ownership | Authority and accountability for organizations | Governance | Ownership |  |
| Alter/improve care pathways | Care coordination | Health service delivery | Care pathways |  |
| Alter/improve communication between providers | Care coordination | Health service delivery | Communication between providers |  |
| Assign case manager | Care coordination | Health service delivery | Case management |  |
| Coordinate care among different providers | Care coordination | Health service delivery | Coordination of care amongst different provider |  |
| Create a multidisciplinary team | Care coordination | Health service delivery | Teams |  |
| Facilitate transition of care  between providers/services | Care coordination | Health service delivery | Transition of Care |  |
| Institute new/altered discharge and/or referral systems | Care coordination | Health service delivery | Discharge planning |  |
| Institute or improve procurement and storage systems | Care coordination | Health service delivery | Procurement and distribution of supplies |  |
| Promote continuity of provider care | Care coordination | Health service delivery | Continuity of care |  |
| Provide multiple types of services by one provider | Care coordination | Health service delivery | Role expansion or task shifting | Revise professional roles |
| Share care between  interdisciplinary teams | Care coordination | Health service delivery | Shared care |  |
| Change staffing models | Human resource strategies | Health service delivery | Staffing models |  |
| Conduct exit interviews | Human resource strategies | Health service delivery | Exit interviews |  |
| Create new clinical teams | Human resource strategies | Health service delivery | Teams | Create new clinical teams |
| Manage staff movement between public and private care | Human resource strategies | Health service delivery | Movement of health workers between public and private care |  |
| Recruit and retain staff | Human resource strategies | Health service delivery | Recruitment and retention strategies for district health managers - low- and middle-income countries |  |
| Use lower cadre of staff to provide services | Human resource strategies | Health service delivery | Role expansion or task shifting | Revise professional roles |
| Institute or improve adverse events reporting | Information and technology | Health service delivery | Clinical incident reporting | Change record systems |
| Introduce/alter health information systems | Information and technology | Health service delivery | Health information systems | Change record systems |
| Provide telemedicine consultation/s | Information and technology | Health service delivery |  |  |
| Send messages about a service/innovation/behaviour | Information and technology | Health service delivery |  | Use mass media |
| Use data warehousing techniques | Information and technology | Health service delivery |  | Use data warehousing techniques |
| Use of information and communication technology | Information and technology | Health service delivery | The use of information and communication technology |  |
| Use technology to provide real-time support | Information and technology | Health service delivery |  |  |
| Change physical structure and equipment | Location and environment of health services | Health service delivery | Environment | Change physical structure and equipment |
| Provide community-based services | Location and environment of health services | Health service delivery | Site of service delivery | Change service sites |
| Provide outreach services | Location and environment of health services | Health service delivery | Outreach services | Change service sites |
| Provide services at lower-level facilities | Location and environment of health services | Health service delivery | Site of service delivery | Change service sites |
| Provide services at private/confidential locations | Location and environment of health services | Health service delivery | Site of service delivery | Change service sites |
| Provide multiple service types at the same location | Location and environment of health services | Health service delivery | Integration | Change service sites |
| Access social or sexual networks | People centred health services | Health service delivery |  |  |
| Offer a choice of services | People centred health services | Health service delivery |  |  |
| Provide couple-based health services | People centred health services | Health service delivery | Group versus individual care | Involve patients/consumers and family members |
| Provide family-based health services | People centred health services | Health service delivery | Group versus individual care | Involve patients/consumers and family members |
| Provide group-based health services | People centred health services | Health service delivery | Group versus individual care |  |
| Provide individualised health services | People centred health services | Health service delivery | Group versus individual care |  |
| Remove administrative requirements to receive care | People centred health services | Health service delivery | Clinical Practice Guidelines |  |
| Trace lost/late patient | People centred health services | Health service delivery |  |  |
| Triage sick patient | People centred health services | Health service delivery | Triage |  |
| Use contact tracing | People centred health services | Health service delivery |  |  |
| Alter sequence of services provided | Timing/duration of health services | Health service delivery |  |  |
| Expedite services | Timing/duration of health services | Health service delivery | Patient-initiated appointment systems |  |
| Extend hours of operation | Timing/duration of health services | Health service delivery | Queuing strategies |  |
| Increase duration of services | Timing/duration of health services | Health service delivery | Queuing strategies |  |
| Increase frequency of services | Timing/duration of health services | Health service delivery | Queuing strategies |  |
| Increase the quantity of medication dispensed | Timing/duration of health services | Health service delivery |  | Intervene with patients/consumers to enhance uptake and adherence |
| Offer unscheduled appointments | Timing/duration of health services | Health service delivery | Patient-initiated appointment systems | Prepare patients/consumers to be active participants |
| Reduce duration of services | Timing/duration of health services | Health service delivery | Queuing strategies |  |
| Reduce frequency of health services | Timing/duration of health services | Health service delivery | Queuing strategies |  |
| Assess health service readiness for change | Adaptation to context | Implementation process |  | Assess for readiness and identify barriers and facilitators |
| Conduct local needs assessment | Adaptation to context | Implementation process | Tailored interventions | Conduct local needs assessment |
| Involve stakeholders in strategy design | Adaptation to context | Implementation process | Local consensus processes | Conduct local consensus discussions |
| Promote adaptability of health innovation | Adaptation to context | Implementation process |  | Promote adaptability |
| Tailor strategies to address barriers and facilitators | Adaptation to context | Implementation process | Tailored interventions | Tailor strategies |
| Capture and share local knowledge | Collaborative and networking approaches | Implementation process |  | Capture and share local knowledge |
| Create a learning collaborative | Collaborative and networking approaches | Implementation process | Communities of practice | Create a learning collaborative |
| Develop academic partnerships | Collaborative and networking approaches | Implementation process |  | Develop academic partnerships |
| Inform local opinion leaders | Collaborative and networking approaches | Implementation process | Local opinion leaders | Inform local opinion leaders |
| Involve executive boards | Collaborative and networking approaches | Implementation process |  | Involve executive boards |
| Obtain formal commitments | Collaborative and networking approaches | Implementation process |  | Obtain formal commitments |
| Organise clinician implementation team meetings | Collaborative and networking approaches | Implementation process |  | Organize clinician implementation team meetings |
| Promote multi-institutional collaboration | Collaborative and networking approaches | Implementation process | Communities of practice | Promote network weaving |
| Promote shared decision making | Collaborative and networking approaches | Implementation process | Shared decision-making | Conduct local consensus discussions |
| Use advisory boards and workgroups | Collaborative and networking approaches | Implementation process |  | Use advisory boards and workgroups |
| Use community mobilization | Collaborative and networking approaches | Implementation process | Community mobilization | Involve patients/consumers and family members |
| Use leaders/champions/influencers to mandate change | Collaborative and networking approaches | Implementation process | Local opinion leaders | Identify and prepare champions |
| Visit other sites | Collaborative and networking approaches | Implementation process |  | Visit other sites |
| Develop a formal implementation blueprint | Implementation planning and execution | Implementation process |  | Develop a formal implementation blueprint |
| Stage implementation scale up | Implementation planning and execution | Implementation process |  | Stage implementation scale up |
| Collect and review consumer feedback | Quality improvement | Implementation process | Patient-mediated interventions | Obtain and use patients/consumers and family feedback |
| Conduct cyclical small tests of change | Quality improvement | Implementation process | Continuous quality improvement | Conduct cyclical small tests of change |
| Conduct performance audit and feedback exercises | Quality improvement | Implementation process | Audit and feedback | Audit and provide feedback |
| Establish quality and safety systems | Quality improvement | Implementation process | Quality and safety systems | Develop and organize quality monitoring systems |
| Evaluate health system process for quality improvement | Quality improvement | Implementation process | Monitoring the performance of the delivery of healthcare | Develop and implement tools for quality monitoring |
| Institute quality improvement processes | Quality improvement | Implementation process | Continuous quality improvement | Develop and organize quality monitoring systems |
| Measure and report clinical/laboratory outcomes | Quality improvement | Implementation process |  |  |
| Measure and report patient-reported outcomes | Quality improvement | Implementation process | Routine patient-reported outcome measures |  |
| Public release of performance data | Quality improvement | Implementation process | Public release of performance data |  |
| Regulate authority and accountability for quality of practice | Quality improvement | Implementation process | Authority and accountability for quality of practice | Develop and organize quality monitoring systems |
