# Supplementary material for: Implementation strategies to improve HIV care cascade outcomes in low‐ and middle‐income countries: a systematic review from 2014 to 2021
Source: J Int AIDS Soc. 2024 Jul 5;27(Suppl 1):e26263. doi: 10.1002/jia2.26263 (PMC11224579; doi:10.1002/jia2.26263)
Supplement: Supplementary file 4 — Supporting information 4. Main and related studies included in the Living Database of HIV Implementation Science (LIVE) systematic review [file JIA2-27-e26263-s003.docx]

**Appendix 4.** Main and related studies included in the Living Database of HIV Implementation Science (LIVE) systematic review of HIV implementation strategies used to improve the HIV care cascade outcomes in low- and middle-income countries, 2014-2021.

| **First author** | **Publication year** | | **Full study title** | **Study design** | **Country** | **Other related studies** |
| --- | --- | --- | --- | --- | --- | --- |
| Ansa | 2014 | Delivering TB/HIV services in Ghana: a comparative study of service delivery models | | Quasi-experimental | Ghana |  |
| Arrivillaga | 2014 | The IMEA project: an intervention based on microfinance, entrepreneurship, and adherence to treatment for women with HIV/AIDS living in poverty | | Quasi-experimental | Colombia |  |
| Asiimwe | 2014 | Accuracy of Un-supervised Versus Provider-Supervised Self-administered HIV Testing in Uganda: A Randomized Implementation Trial | | Randomized controlled | Uganda |  |
| Bassett | 2014 | Linkage to care following community-based mobile HIV testing compared to clinic-based testing in Umlazi Township, Durban, South Africa | | Cohort | South Africa |  |
| Bigna | 2014 | Effect of mobile phone reminders on follow-up medical care of children exposed to or infected with HIV in Cameroon (MORE CARE): a multicentre, single-blind, factorial, randomised controlled trial | | Randomized controlled | Cameroon |  |
| Bigogo | 2014 | The impact of home-based HIV counseling and testing on care-seeking and incidence of common infectious disease syndromes in rural western Kenya | | Quasi-experimental | Kenya |  |
| Bindoria | 2014 | Development and pilot testing of HIV screening program integration within public/primary health centers providing antenatal care services in Maharashtra, India | | Quasi-experimental | India |  |
| Black | 2014 | Improving HIV testing amongst adolescents through an integrated Youth Centre rewards program: Insights from South Africa | | Cohort | South Africa |  |
| Clouse | 2014 | Impact of systematic HIV testing on case finding and retention in care at a primary care clinic in South Africa | | Cohort | South Africa |  |
| Coates | 2014 | Effect of community-based voluntary counselling and testing on HIV incidence and social and behavioural outcomes (NIMH Project Accept; HPTN 043): a cluster-randomised trial | | Randomized controlled | South Africa, Tanzania, Thailand, Zimbabwe |  |
| Das | 2014 | Retention Among ART Patients in the Highlands of Papua New Guinea: Evaluating the PAPUA Model | | Randomized controlled | Papua New Guinea |  |
| Decroo | 2014 | Four-year retention and risk factors for attrition among members of community ART groups in Tete, Mozambique | | Cohort | Mozambique |  |
| Djarma | 2014 | Continuous free access to HAART could be one of the potential factors impacting on loss to follow-up in HAART-eligible patients living in a resource-limited setting: N'djamena, Chad | | Cohort | Chad |  |
| Fatti | 2014 | Improved virological suppression in children on antiretroviral treatment receiving community based adherence support: A multicentre cohort study from South Africa | | Cohort | South Africa |  |
| Finocchario-Kessler | 2014 | If you text them, they will come: using the HIV infant tracking system to improve early infant diagnosis quality and retention in Kenya | | Quasi-experimental | Kenya |  |
| Galindo-Quintero | 2014 | HIV Testing and Counselling in Colombia: Local Experience on Two Different Recruitment Strategies to Better Reach Low Socioeconomic Status Communities | | Program evaluation | Colombia |  |
| Gerdts | 2014 | Linkage to HIV Care and Antiretroviral Therapy by HIV Testing Service Type in Central Mozambique: A Retrospective Cohort Study | | Cohort | Mozambique |  |
| Grimsrud | 2014 | Outcomes of a nurse-managed service for stable HIV-positive patients in a large South African public sector antiretroviral therapy programme | | Cohort | South Africa |  |
| Ikeda | 2014 | Impact of Integrating HIV and TB Care and Treatment in a Regional Tuberculosis Hospital in Rural Guatemala | | Cross-sectional | Guatemala |  |
| Jain | 2014 | Successful Antiretroviral Therapy Delivery and Retention in Care Among Asymptomatic Individuals with High CD4+ T Cell Counts ≥350 cells/uL in Rural Uganda | | Program evaluation | Uganda |  |
| Kaihin | 2014 | Effect of an Empowerment Intervention on Antiretroviral Drug Adherence in Thai Youth | | Cohort | Thailand |  |
| Kaufman | 2014 | AIDS Impact/SEISIDA Special Issue: Using Social and Behavior Change Communication to Increase HIV Testing and Condom Use: The Malawi BRIDGE Project | | Cohort | Malawi |  |
| Kikaya | 2014 | Voluntary Medical Male Circumcision Programs Can Address Low HIV Testing and Counseling Usage and ART Enrollment among Young Men: Lessons from Lesotho | | Cohort | Lesotho |  |
| Kyegombe | 2014 | The impact of SASA!, a community mobilization intervention, on reported HIV-related risk behaviours and relationship dynamics in Kampala, Uganda | | Randomized controlled | Uganda |  |
| Labhardt | 2014 | Home-Based Versus Mobile Clinic HIV Testing and Counseling in Rural Lesotho: A Cluster-Randomized Trial | | Randomized controlled | Lesotho |  |
| Leon | 2014 | A comparison of linkage to HIV care after provider-initiated HIV testing and counselling (PITC) versus voluntary HIV counselling and testing (VCT) for patients with sexually transmitted infections in Cape Town, South Africa | | Cohort | South Africa |  |
| MacPherson | 2014 | Effect of Optional Home Initiation of HIV Care Following HIV Self-testing on Antiretroviral Therapy Initiation Among Adults in Malawi | | Randomized controlled | Malawi |  |
| Martinez | 2014 | Tailored Nutrition Education and Food Assistance Improve Adherence to HIV Antiretroviral Therapy: Evidence from Honduras | | Randomized controlled | Honduras |  |
| Obua | 2014 | Improving adherence to antiretroviral treatment in Uganda with a low-resource facility-based intervention | | Cohort | Uganda |  |
| Odeny | 2014 | Texting improves testing: a randomized trial of two-way SMS to increase postpartum prevention of mother-to-child transmission retention and infant HIV testing | | Randomized controlled | Kenya |  |
| Oluoch | 2014 | Electronic medical record systems are associated with appropriate placement of HIV patients on antiretroviral therapy in rural health facilities in Kenya: a retrospective pre-post study | | Cohort | Kenya |  |
| Osoti | 2014 | Home Visits during Pregnancy Enhance Male Partner HIV Counseling and Testing in Kenya: A Randomized Clinical Trial | | Randomized controlled | Kenya |  |
| OʼLaughlin | 2014 | Clinic-based routine voluntary HIV testing in a refugee settlement in Uganda | | Cohort | Uganda |  |
| Reidy | 2014 | Decentralization of HIV Care and Treatment Services in Central Province, Kenya | | Cohort | Kenya |  |
| Shet | 2014 | Effect of mobile telephone reminders on treatment outcome in HIV: evidence from a randomised controlled trial in India | | Randomized controlled | India |  |
| Snyder | 2014 | Preliminary results from Hlanganani (Coming Together): A structured support group for HIV-infected adolescents piloted in Cape Town, South Africa | | Cohort | South Africa |  |
| Solomon | 2014 | Voucher Incentives Improve Linkage to and Retention in Care Among HIV-Infected Drug Users in Chennai, India | | Randomized controlled | India |  |
| van Dijk | 2014 | Scaling-Up Access to Antiretroviral Therapy for Children: A Cohort Study Evaluating Care and Treatment at Mobile and Hospital-Affiliated HIV Clinics in Rural Zambia | | Cohort | Zambia |  |
| van Lettow | 2014 | Towards elimination of mother-to-child transmission of HIV: performance of different models of care for initiating lifelong antiretroviral therapy for pregnant women in Malawi (Option B) | | Cohort | Malawi |  |
| Van Rie | 2014 | Integration and Task Shifting for TB/HIV Care and Treatment in Highly Resource Scarce-Settings: One Size May Not Fit All | | Cohort | Democratic Republic of the Congo |  |
| Wagman | 2014 | Effectiveness of an integrated intimate partner violence and HIV prevention intervention in Rakai, Uganda: analysis of an intervention in an existing cluster randomised cohort. | | Randomized controlled | Uganda |  |
| Weaver | 2014 | Improving Facility Performance in Infectious Disease Care in Uganda: A Mixed Design Study with Pre/Post and Cluster Randomized Trial Components | | Randomized controlled | Uganda |  |
| Williams | 2014 | Efficacy of an Evidence-Based ARV Adherence Intervention in China | | Randomized controlled | People's Republic of China |  |
| Winter | 2014 | Evaluation of an HIV Adherence Counseling Program in La Romana, Dominican Republic | | Cohort | Dominican Republic |  |
| Yan | 2014 | A peer-led, community-based rapid HIV testing intervention among untested men who have sex with men in China: an operational model for expansion of HIV testing and linkage to care | | Cohort | People's Republic of China |  |
| Zeng | 2014 | Efficiency of HIV/AIDS Health Centers and Effect of Community-Based Health Insurance and Performance-Based Financing on HIV/AIDS Service Delivery in Rwanda | | Cohort | Rwanda |  |
| Zhou | 2014 | Treatment Adherence and Health Outcomes in MSM with HIV/AIDS: Patients Enrolled in ‘‘One-Stop’’ and Standard Care Clinics in Wuhan China | | Program evaluation | People's Republic of China |  |
| Ahmed | 2015 | Improved identification and enrolment into care of HIV-exposed and -infected infants and children following a community health worker intervention in Lilongwe, Malawi | | Cohort | Malawi |  |
| Anand | 2015 | Innovative strategies using communications technologies to engage gay men and other men who have sex with men into early HIV testing and treatment in Thailand | | Cohort | Thailand |  |
| Aninanya | 2015 | Effects of an Adolescent Sexual and Reproductive Health Intervention on Health Service Usage by Young People in Northern Ghana: A Community-Randomised Trial | | Randomized controlled | Ghana |  |
| Bachireddy | 2015 | Integration of Health Services Improves Multiple Healthcare Outcomes Among HIV-infected People Who Inject Drugs in Ukraine | | Cross-sectional | Ukraine |  |
| Bassett | 2015 | Linkage to care following community‐based mobile HIV testing compared with clinic‐based testing in Umlazi Township, Durban, South Africa | | Cohort | South Africa |  |
| Chang | 2015 | Effectiveness of Peer Support on Care Engagement and Preventive Care Intervention Utilization Among Pre-antiretroviral Therapy, HIV-Infected Adults in Rakai, Uganda: A Randomized Trial | | Randomized controlled | Uganda |  |
| de Walque | 2015 | Using provider performance incentives to increase HIV testing and counseling services in Rwanda | | Randomized controlled | Rwanda |  |
| Dryden-Peterson | 2015 | An Augmented SMS Intervention to Improve Access to Antenatal CD4 Testing and ART Initiation in HIV-Infected Pregnant Women: A Cluster Randomized Trial | | Randomized controlled | Botswana |  |
| Fayorsey | 2015 | Decentralization of Pediatric HIV Care and Treatment in Five Sub-Saharan African Countries | | Cohort | Kenya, Lesotho, Mozambique, Rwanda, Tanzania | |
| Ferrand | 2015 | Implementation and Operational Research: The Effectiveness of Routine Opt-Out HIV Testing for Children in Harare, Zimbabwe | | Case-control | Zimbabwe |  |
| Girault | 2015 | Piloting a Social Networks Strategy to Increase HIV Testing and Counseling Among Men Who Have Sex with Men in Greater Accra and Ashanti Region, Ghana | | Program evaluation | Ghana |  |
| Grimsrud | 2015 | Implementation of community-based adherence clubs for stable antiretroviral therapy patients in Cape Town, South Africa | | Cohort | South Africa |  |
| Haskew | 2015 | Implementation of a Cloud-Based Electronic Medical Record to Reduce Gaps in the HIV Treatment Continuum in Rural Kenya | | Quasi-experimental | Kenya |  |
| Herlihy | 2015 | Integration of PMTCT and Antenatal Services Improves Combination Antiretroviral Therapy Uptake for HIV-Positive Pregnant Women in Southern Zambia: A Prototype for Option B+? | | Quasi-experimental | Zambia |  |
| Himmich | 2015 | Scaling up combined community-based HIV prevention interventions targeting truck drivers in Morocco: effectiveness on HIV testing and counseling | | Quasi-experimental | Morocco |  |
| Kimani | 2015 | Use of HIV counseling and testing and family planning services among postpartum women in Kenya: a multicentre, non-randomised trial | | Cohort | Kenya |  |
| LaCourse | 2015 | Implementation of routine counselor-initiated opt-out HIV testing on the adult medical ward at Kamuzu Central Hospital, Lilongwe, Malawi | | Quasi-experimental | Malawi |  |
| Manumbu | 2015 | Shortening Turnaround Times for Newborn HIV Testing in Rural Tanzania: A Report from the Field | | Case-control | Tanzania |  |
| McNaghten | 2015 | Strengthening HIV Test Access and Treatment Uptake Study (Project STATUS): A Randomized Trial of HIV Testing and Counseling Interventions | | Randomized controlled | South Africa, Tanzania, Uganda |  |
| Mi | 2015 | Effects of a Quasi-Randomized Web-Based Intervention on Risk Behaviors and Treatment Seeking Among HIV-Positive Men Who Have Sex With Men in Chengdu, China | | Quasi-experimental | People's Republic of China |  |
| Nakigozi | 2015 | Impact of Patient-Selected Care Buddies on Adherence to HIV Care, Disease Progression, and Conduct of Daily Life Among Pre-antiretroviral HIV-Infected Patients in Rakai, Uganda: A Randomized Controlled Trial | | Randomized controlled | Uganda |  |
| Namukwaya | 2015 | Use of peers, community lay persons and Village Health Team (VHT) members improves six-week postnatal clinic (PNC) follow-up and Early Infant HIV Diagnosis (EID) in urban and rural health units in Uganda: A one-year implementation study | | Cohort | Uganda |  |
| Nyondo | 2015 | Invitation Cards during Pregnancy Enhance Male Partner Involvement in Prevention of Mother to Child Transmission (PMTCT) of Human Immunodeficiency Virus (HIV) in Blantyre, Malawi: A Randomized Controlled Open Label Trial | | Randomized controlled | Malawi |  |
| Robbins | 2015 | Enhancing Lay Counselor Capacity to Improve Patient Outcomes with Multimedia Technology | | Randomized controlled | South Africa |  |
| Sabin | 2015 | Improving Adherence to Antiretroviral Therapy with Triggered Real Time Text Message Reminders: the China through Technology Study (CATS) | | Randomized controlled | People's Republic of China |  |
| Siedner | 2015 | A combination SMS and transportation reimbursement intervention to improve HIV care following abnormal CD4 test results in rural Uganda: a prospective observational cohort study | | Cohort | Uganda |  |
| Turan | 2015 | Effects of antenatal care and HIV treatment integration on elements of the PMTCT cascade: Results from the SHAIP cluster-randomized controlled trial in Kenya | | Randomized controlled | Kenya |  |
| van Loggerenberg | 2015 | Individualised Motivational Counselling to Enhance Adherence to Antiretroviral Therapy is not Superior to Didactic Counselling in South African Patients: Findings of the CAPRISA 058 Randomised Controlled Trial | | Randomized controlled | South Africa |  |
| Vogt | 2015 | Tracing defaulters in HIV prevention of mother-to-child transmission programmes through community health workers: results from a rural setting in Zimbabwe | | Cohort | Zimbabwe |  |
| Wang | 2015 | A Cluster Randomised Trial on the Impact of Integrating Early Infant HIV Diagnosis with the Expanded Programme on Immunization on Immunization and HIV Testing Rates in Rural Health Facilities in Southern Zambia | | Randomized controlled | Zambia |  |
| Washington | 2015 | The effect of integration of HIV care and treatment into antenatal care clinics on mother-to-child HIV transmission and maternal outcomes in Nyanza, Kenya: results from the SHAIP cluster randomized controlled trial | | Randomized controlled | Kenya |  |
| Wu | 2015 | Simplified HIV Testing and Treatment in China: Analysis of Mortality Rates Before and After a Structural Intervention | | Quasi-experimental | People's Republic of China |  |
| Zeng | 2015 | Exploring HIV Prevention Strategies among Street-Based Female Sex Workers in Chongqing, China | | Quasi-experimental | People's Republic of China |  |
| Aderemi-Williams | 2016 | Effect of 2 Models of Care and Factors Predicting Patients’ Adherence to Doctor’s Appointment Attendance in Lagos State University Teaching Hospital, Ikeja, Lagos, Nigeria | | Quasi-experimental | Nigeria |  |
| Ahmed | 2016 | Lost opportunities to identify and treat HIV‐positive patients: results from a baseline assessment of provider‐initiated HIV testing and counselling (PITC) in Malawi | | Cross-sectional | Malawi |  |
| Aliyu | 2016 | Integrated prevention of mother-to-child HIV transmission services, antiretroviral therapy initiation, and maternal and infant retention in care in rural north-central Nigeria: a cluster-randomised controlled trial | | Randomized controlled | Nigeria |  |
| Amanyire | 2016 | Effects of a multicomponent intervention to streamline initiation of antiretroviral therapy in Africa: a stepped-wedge cluster-randomised trial | | Randomized controlled | Uganda |  |
| Atanga | 2016 | Retention in care and reasons for discontinuation of lifelong antiretroviral therapy in a cohort of Cameroonian pregnant and breastfeeding HIV‐positive women initiating ‘Option B+’ in the South West Region | | Cohort | Cameroon |  |
| Audet | 2016 | Engagement of men in antenatal care services: Increased HIV testing and treatment uptake in a community participatory action program in Mozambique | | Quasi-experimental | Mozambique |  |
| Auld | 2016 | Decentralizing Access to Antiretroviral Therapy for Children Living with HIV in Swaziland. | | Cohort | eSwatini | 10.1097/QAI.0000000000000547 |
| Bango | 2016 | Adherence clubs for long-term provision of antiretroviral therapy: cost-effectiveness and access analysis from Khayelitsha, South Africa | | Cross-sectional | South Africa |  |
| Barnabas | 2016 | Uptake of antiretroviral therapy and male circumcision following community-based HIV testing linkage strategies versus referral: A randomized, multisite, open-label, individual trial in South Africa and Uganda | | Randomized controlled | South Africa, Uganda |  |
| Bassett | 2016 | Sizanani: A Randomized Trial of Health System Navigators to Improve Linkage to HIV and TB Care in South Africa | | Randomized controlled | South Africa |  |
| Booth | 2016 | HIV incidence among people who inject drugs (PWID) in Ukraine: results from a clustered randomized trial | | Randomized controlled | Ukraine |  |
| Breger | 2016 | Self-disclosure of HIV status, disclosure counseling, and retention in HIV care in Cameroon | | Cohort | Cameroon |  |
| Brunie | 2016 | Expanding HIV testing and counselling into communities: Feasibility, acceptability, and effects of an integrated family planning/HTC service delivery model by Village Health Teams in Uganda | | Randomized controlled | Uganda |  |
| Chan | 2016 | Same day HIV diagnosis and antiretroviral therapy initiation affects retention in Option B prevention of mother-to-child transmission services at antenatal care in Zomba District, Malawi | | Cohort | Malawi |  |
| Choko | 2016 | HIV self-testing alone or with additional interventions, including financial incentives, and linkage to care or prevention among male partners of antenatal care clinic attendees in Malawi: An adaptive, multi-arm, multi-stage cluster randomised trial | | Randomized controlled | Malawi |  |
| Colchero | 2016 | Impact and economic evaluations of a combination prevention programme for men who have sex with men in Mexico | | Cohort | Mexico |  |
| Cuong | 2016 | Impact of peer support on virologic failure in HIV-infected patients on antiretroviral therapy - a cluster randomized controlled trial in Vietnam | | Randomized controlled | Vietnam |  |
| Fatti | 2016 | Improved long-term antiretroviral treatment outcomes amongst patients receiving community-based adherence support in South Africa | | Cohort | South Africa |  |
| Fatti | 2016 | A Comparison of Two Task-Shifting Models of Pharmaceutical Care in Antiretroviral Treatment Programs in South Africa | | Cohort | South Africa |  |
| Ferrand | 2016 | The Effectiveness of Routine Opt-Out HIV Testing for Children in Harare, Zimbabwe | | Quasi-experimental | Zimbabwe |  |
| Gamell | 2016 | An Integrated and Comprehensive Service Delivery Model to Improve Pediatric and Maternal HIV Care in Rural Africa | | Cohort | Tanzania |  |
| Glasman | 2016 | Using Peer-Referral Chains with Incentives to Promote HIV Testing and Identify Undiagnosed HIV Infections Among Crack Users in San Salvador | | Quasi-experimental | El Salvador |  |
| Grimsrud | 2016 | Community-Based Adherence Clubs for the Management of Stable Antiretroviral Therapy Patients in Cape Town, South Africa: A Cohort Study | | Cohort | South Africa |  |
| Gupta | 2016 | Use of technology in follow-up of HIV positive pregnant women and their babies till 18 months of age- an innovation by Maharashtra State AIDS Control Society (MSACS), India | | Quasi-experimental | India |  |
| Haberer | 2016 | Short message service (SMS) reminders and real-time adherence monitoring improve antiretroviral therapy adherence in rural Uganda | | Randomized controlled | Uganda |  |
| Hewett | 2016 | Randomized evaluation and cost-effectiveness of HIV and sexual and reproductive health service referral and linkage models in Zambia | | Randomized controlled | Zambia |  |
| Huang | 2016 | Assessment of Different Intervention Models of Male Circumcision and Their Preliminary Effectiveness in Reducing HIV Incidence Among Drug Users in Western China | | Randomized controlled | People's Republic of China |  |
| Jobarteh | 2016 | Community ART Support Groups in Mozambique: The Potential of Patients as Partners in Care | | Cohort | Mozambique |  |
| Johns | 2016 | The Cost-Effectiveness of Integrating HIV Counseling and Testing into Primary Health Care in the Ukraine | | Quasi-experimental | Ukraine |  |
| Jones | 2016 | Improving adherence to care among “hard to reach” HIV-infected patients in Argentina | | Quasi-experimental | Argentina |  |
| Joseph Davey | 2016 | SMSaúde: Evaluating mobile phone text reminders to improve retention in HIV care for patients on antiretroviral therapy in Mozambique | | Randomized controlled | Mozambique |  |
| Kaplan | 2016 | An integrated community TB-HIV adherence model provides an alternative to DOT for tuberculosis patients in Cape Town | | Cohort | South Africa |  |
| Kassaye | 2016 | Cluster-Randomized Controlled Study of SMS Text Messages for Prevention of Mother-to-Child Transmission of HIV in Rural Kenya | | Randomized controlled | Kenya |  |
| Kemp | 2016 | Mixed-Methods Evaluation of a Novel, Structured, Community- Based Support and Education Intervention for Individuals with HIV/AIDS in KwaZulu-Natal, South Africa | | Quasi-experimental | South Africa |  |
| Kerrigan | 2016 | Abriendo Puertas: Feasibility and Effectiveness a Multi-Level Intervention to Improve HIV Outcomes Among Female Sex Workers Living with HIV in the Dominican Republic | | Cohort | Dominican Republic |  |
| Kiragga | 2016 | Impact of nurse-targeted care on HIV outcomes among immunocompromised persons: a before-after study in Uganda | | Cohort | Uganda |  |
| Krakowiak | 2016 | Home-Based HIV Testing Among Pregnant Couples Increases Partner Testing and Identification of Serodiscordant Partnerships | | Randomized controlled | Kenya |  |
| Masters | 2016 | Promoting Partner Testing and Couples Testing through Secondary Distribution of HIV Self-Tests: A Randomized Clinical Trial | | Randomized controlled | Kenya |  |
| Matovu | 2016 | Evaluation of a demand-creation intervention for couples’ HIV testing services among married or cohabiting individuals in Rakai, Uganda: a cluster-randomized intervention trial | | Randomized controlled | Uganda |  |
| McGovern | 2016 | Do gifts increase consent to home-based HIV testing? A difference-in-differences study in rural KwaZulu-Natal, South Africa | | Quasi-experimental | South Africa |  |
| Mugo | 2016 | Effect of Text Message, Phone Call, and In-Person Appointment Reminders on Uptake of Repeat HIV Testing among Outpatients Screened for Acute HIV Infection in Kenya: A Randomized Controlled Trial | | Randomized controlled | Kenya |  |
| Muhula | 2016 | Uptake and linkage into care over one year of providing HIV testing and counselling through community and health facility testing modalities in urban informal settlement of Kibera, Nairobi Kenya | | Cohort | Kenya |  |
| Myers | 2016 | Acceptability and Effectiveness of Assisted Human Immunodeficiency Virus Partner Services in Mozambique: Results From a Pilot Program in a Public, Urban Clinic | | Cohort | Mozambique |  |
| Njuguna | 2016 | The Effect of Human Immunodeficiency Virus Prevention and Reproductive Health Text Messages on Human Immunodeficiency Virus Testing Among Young Women in Rural Kenya | | Quasi-experimental | Kenya |  |
| Nsagha | 2016 | A Randomized Controlled Trial on the Usefulness of Mobile Text Phone Messages to Improve the Quality of Care of HIV and AIDS Patients in Cameroon | | Randomized controlled | Cameroon |  |
| Oyewale | 2016 | The use of vouchers in HIV prevention, referral treatment, and care for young MSM and young transgender people in Dhaka, Bangladesh: experience from ‘HIM’ initiative | | Cross-sectional | Bangladesh |  |
| Pascom | 2016 | Point-of-care HIV tests done by peers, Brazil | | Cohort | Brazil |  |
| Pettifor | 2016 | The effect of a conditional cash transfer on HIV incidence in young women in rural South Africa (HPTN 068): a phase 3, randomised controlled trial | | Randomized controlled | South Africa |  |
| Rachlis | 2016 | Evaluating outcomes of patients lost to follow-up in a large comprehensive care treatment program in western Kenya | | Cohort | Kenya |  |
| Reif | 2016 | Impact of a youth-friendly HIV clinic: 10 years of adolescent outcomes in Port-au-Prince, Haiti. | | Cohort | Haiti | 10.1089/apc.2016.0102 |
| Rosen | 2016 | Initiating Antiretroviral Therapy for HIV at a Patient's First Clinic Visit: The RapIT Randomized Control Trial | | Randomized controlled | South Africa | 10.1097/qad.0000000000001528 |
| Tang | 2016 | Crowdsourcing HIV Test Promotion Videos: A Non-Inferiority Randomized Controlled Trial in China | | Randomized controlled | People's Republic of China |  |
| Thirumurthy | 2016 | Promoting male partner testing and safer sexual decision-making through secondary distribution of HIV self-tests by HIV-uninfected female sex workers and women receiving antenatal and postpartum care in Kenya: a cohort study | | Cohort | Kenya |  |
| Yotebieng | 2016 | Conditional cash transfers and uptake of and retention in prevention of mother-to-child HIV transmission care: a randomised controlled trial | | Randomized controlled | Democratic Republic of the Congo |  |
| Yotebieng | 2016 | Provider-Initiated HIV testing and counseling among patients with presumptive tuberculosis in Democratic Republic of Congo | | Cohort | Democratic Republic of the Congo |  |
| Abdulrahman | 2017 | Mobile phone reminders and peer counseling improve adherence and treatment outcomes of patients on ART in Malaysia: A randomized clinical trial | | Randomized controlled | Malaysia |  |
| Akinleye | 2017 | Integration of HIV Testing into Maternal, Newborn, and Child Health Weeks for Improved Case Finding and Linkage to Prevention of Mother-to-Child Transmission Services in Benue State, Nigeria | | Cross-sectional | Nigeria |  |
| Aung | 2017 | Effectiveness of an Integrated Community- and Clinic-Based Intervention on HIV Testing, HIV Knowledge, and Sexual Risk Behavior of Young Men Who Have Sex With Men in Myanmar | | Quasi-experimental | Myanmar |  |
| Bekolo | 2017 | Six-monthly appointment spacing for clinical visits as a model for retention in HIV Care in Conakry-Guinea: a cohort study | | Cohort | Guinea |  |
| Bello | 2017 | The effect of engaging unpaid informal providers on case detection and treatment initiation rates for TB and HIV in rural Malawi (Triage Plus): A cluster randomised health system intervention trial | | Randomized controlled | Malawi |  |
| Bezabih | 2017 | Comparison of treatment adherence outcome among PLHIV enrolled in economic strengthening program with community control | | Cross-sectional | Ethiopia |  |
| Bilinski | 2017 | Distance to care, enrollment and loss to follow-up of HIV patients during decentralization of antiretroviral therapy in Neno District, Malawi: A retrospective cohort study | | Cohort | Malawi |  |
| Bogart | 2017 | A Comparison of Home-Based Versus Outreach Event-Based Community HIV Testing in Ugandan Fisherfolk Communities | | Case-control | Uganda |  |
| Broughton | 2017 | Cost-effectiveness of implementing the chronic care model for HIV care in Uganda | | Quasi-experimental | Uganda |  |
| CarolineRuria | 2017 | Optimizing linkage to care and initiation and retention on treatment of adolescents with newly diagnosed HIV infection | | Quasi-experimental | Kenya |  |
| Chanda | 2017 | HIV self-testing among female sex workers in Zambia: A cluster randomized controlled trial | | Randomized controlled | Zambia |  |
| Cherutich | 2017 | Assisted partner services for HIV in Kenya: a cluster randomized control trial | | Randomized controlled | Kenya | 10.1097/QAI.0000000000001638 |
| Chizoba | 2017 | Increasing HIV Testing Among Pregnant Women in Nigeria: Evaluating the Traditional Birth Attendant and Primary Health Center Integration (TAP-In) Model | | Quasi-experimental | Nigeria |  |
| Decroo | 2017 | Effect of Community ART Groups on retention-in-care among patients on ART in Tete Province, Mozambique: a cohort study | | Cohort | Mozambique |  |
| Desai | 2017 | Effect of point-of-care CD4 cell count results on linkage to care and antiretroviral initiation during a home-based HIV testing campaign: a non-blinded, cluster-randomized trial | | Randomized controlled | Kenya |  |
| Elul | 2017 | A combination intervention strategy to improve linkage to and retention in HIV care following diagnosis in Mozambique: a cluster-randomized trial | | Randomized controlled | Mozambique |  |
| Ezeanolue | 2017 | What do You Need to Get Male Partners of Pregnant Women Tested for HIV in Resource Limited Settings? The Baby Shower Cluster Randomized Trial | | Randomized controlled | Nigeria |  |
| Ferrand | 2017 | The effect of community-based support for caregivers on the risk of virological failure in children and adolescents with HIV in Harare, Zimbabwe (ZENITH): an open-label, randomised controlled trial | | Randomized controlled | Zimbabwe |  |
| Ford | 2017 | Implementation of Antiretroviral Therapy for Life in Pregnant/Breastfeeding HIV+ Women (Option B+) Alongside Rollout and Changing Guidelines for ART Initiation in Rural Zimbabwe: The Lablite Project Experience | | Quasi-experimental | Zimbabwe | 10.1093/inthealth/ihx061, 10.1093/inthealth/ihz090 |
| Foster | 2017 | Impact of facility-based mother support groups on retention in care and PMTCT outcomes in rural Zimbabwe: The EPAZ cluster-randomized controlled trial | | Randomized controlled | Zimbabwe |  |
| Franse | 2017 | Linkage to HIV care before and after the introduction of provider-initiated testing and counselling in six Rwandan health facilities | | Quasi-experimental | Rwanda |  |
| Galárraga | 2017 | Punto Seguro: A Randomized Controlled Pilot Using Conditional Economic Incentives to Reduce Sexually Transmitted Infection Risks in Mexico | | Randomized controlled | Mexico |  |
| Georgette | 2017 | Impact of a clinical program using weekly Short Message Service (SMS) on antiretroviral therapy adherence support in South Africa: a retrospective cohort study | | Cohort | South Africa |  |
| Gerenutti | 2017 | The Effectiveness of a Pharmaceutical Care Model on Adherence to Antiretroviral Therapy: A SAME-Based Cohort Study in Brazil | | Cohort | Brazil |  |
| Go | 2017 | Increased survival among HIV-infected PWID receiving a multi-level HIV risk and stigma reduction intervention: results from a randomized control trial | | Randomized controlled | Vietnam |  |
| Hoffmann | 2017 | Strategies to Accelerate HIV Care and Antiretroviral Therapy Initiation After HIV Diagnosis: A Randomized Trial | | Randomized controlled | South Africa |  |
| Iwuji | 2017 | Universal test and treat and the HIV epidemic in rural South Africa: a phase 4, open-label, community cluster randomised trial | | Randomized controlled | South Africa | 10.1007/s11904-020-00487-1 |
| Joseph | 2017 | Impact of Point-of-Care CD4 Testing on Retention in Care Among HIV-Positive Pregnant and Breastfeeding Women in the Context of Option B+ in Zimbabwe: A Cluster Randomized Controlled Trial | | Randomized controlled | Zimbabwe |  |
| Kelvin | 2017 | Offering self-administered oral HIV testing to truck drivers in Kenya to increase testing: a randomized controlled trial | | Randomized controlled | Kenya |  |
| Koenig | 2017 | Same-day HIV testing with initiation of antiretroviral therapy versus standard care for persons living with HIV: A randomized unblinded trial | | Randomized controlled | Haiti |  |
| Lifson | 2017 | Implementation of a Peer HIV Community Support Worker Program in Rural Ethiopia to Promote Retention in Care | | Cohort | Ethiopia |  |
| Linnemayr | 2017 | Behavioral economic incentives to improve adherence to antiretroviral medication | | Randomized controlled | Uganda |  |
| Linnemayr | 2017 | Text messages for improving antiretroviral adherence: no effects after 1 year among adolescents and young adults | | Randomized controlled | Uganda |  |
| Lippman | 2017 | Community Mobilization for HIV Testing Uptake: Results From a Community Randomized Trial of a Theory-Based Intervention in Rural South Africa | | Randomized controlled | South Africa |  |
| McCoy | 2017 | Cash versus food assistance to improve adherence to antiretroviral therapy among HIV-infected adults in Tanzania: a randomized trial | | Randomized controlled | Tanzania |  |
| McCoy | 2017 | Pilot study of a multi-pronged intervention using social norms and priming to improve adherence to antiretroviral therapy and retention in care among adults living with HIV in Tanzania | | Quasi-experimental | Tanzania |  |
| McNairy | 2017 | Effectiveness of a combination strategy for linkage and retention in adult HIV care in Swaziland: The Link4Health cluster-randomized trial | | Randomized controlled | eSwatini |  |
| McNairy | 2017 | Task-sharing with nurses to enhance access to HIV treatment in Côte d’Ivoire | | Cohort | Ivory Coast |  |
| Mwapasa | 2017 | Impact of Mother–Infant Pair Clinics and Short-Text Messaging Service (SMS) Reminders on Retention of HIV-Infected Women and HIV-Exposed Infants in eMTCT Care in Malawi: A Cluster Randomized Trial | | Randomized controlled | Malawi |  |
| Myer | 2017 | Differentiated models of care for postpartum women on antiretroviral therapy in Cape Town, South Africa: a cohort study | | Cohort | South Africa |  |
| Nance | 2017 | Short-term effectiveness of a community health worker intervention for HIV-infected pregnant women in Tanzania to improve treatment adherence and retention in care: A cluster-randomized trial | | Randomized controlled | Tanzania |  |
| NgaiSze | 2017 | MSM HIV testing following an online testing intervention in China | | Cross-sectional | People's Republic of China |  |
| Ogbo | 2017 | Assessment of provider-initiated HIV screening in Nigeria with sub-Saharan African comparison | | Cohort | Nigeria |  |
| Oluoch | 2017 | Application of psychosocial models to Home-Based Testing and Counseling (HBTC) for increased uptake and household coverage in a large informal urban settlement in Kenya | | Cross-sectional | Kenya |  |
| Ortblad | 2017 | Direct provision versus facility collection of HIV self-tests among female sex workers in Uganda: A cluster-randomized controlled health systems trial | | Randomized controlled | Uganda |  |
| Oyeledun | 2017 | The Effect of a Continuous Quality Improvement Intervention on Retention-In-Care at 6 Months Postpartum in a PMTCT Program in Northern Nigeria: Results of a Cluster Randomized Controlled Study | | Randomized controlled | Nigeria |  |
| Peltzer | 2017 | A cluster randomized controlled trial of lay health worker support for prevention of mother to child transmission of HIV (PMTCT) in South Africa | | Randomized controlled | South Africa |  |
| Pfeiffer | 2017 | Stepped-Wedge Cluster Randomized Controlled Trial to Promote Option B+ Retention in Central Mozambique | | Randomized controlled | Mozambique |  |
| Phiri | 2017 | Impact of Facility- and Community-Based Peer SupportModels on Maternal Uptake and Retention in Malawi’s OptionB+ HIV Prevention of Mother-to-Child Transmission Program: A 3-Arm Cluster Randomized Controlled Trial (PURE Malawi) | | Randomized controlled | Malawi | 10.1097/qai.0000000000001368 |
| Rebeiro | 2017 | An Observational Study of the Effect of Patient Outreach on Return to Care: The Earlier the Better | | Cohort | Kenya |  |
| Ross-Degnan | 2017 | A group randomized trial using an appointment system to improve adherence to ART at reproductive and child health clinics implementing Option B+ in Tanzania | | Randomized controlled | Tanzania |  |
| Rustagi | 2017 | Impact of a systems engineering intervention on PMTCT service delivery in Côte d’Ivoire, Kenya, Mozambique: a cluster randomized trial | | Randomized controlled | Ivory Coast, Kenya, Mozambique | 10.7448/IAS.19.6.21264 |
| Ruzagira | 2017 | Brief counselling after home-based counselling and testing strongly increases linkage to care: a cluster-randomized trial in Uganda | | Randomized controlled | Uganda |  |
| Sam-Agudu | 2017 | The Impact of Structured Mentor Mother Programs on 6-Month Postpartum Retention and Viral Suppression among HIV-Positive Women in Rural Nigeria: A Prospective Paired Cohort Study. | | Cohort | Nigeria |  |
| Sanga | 2017 | Linkage into care among newly diagnosed HIV-positive individuals tested through outreach and facility based HIV testing models in Mbeya, Tanzania: a prospective mixed-method cohort study | | Cohort | Tanzania |  |
| Sarko | 2017 | HIV status disclosure, facility-based delivery and postpartum retention of mothers in a prevention clinical trial in rural Nigeria | | Randomized controlled | Nigeria |  |
| Sebastian | 2017 | Service utilization and cost of implementing a comprehensive HIV prevention and care program among people who inject drugs in Delhi, India | | Economic evaluation | India |  |
| Shenoi | 2017 | Integrated Tuberculosis/Human Immunodeficiency Virus Community-Based Case Finding in Rural South Africa: Implications for Tuberculosis Control Efforts | | Cross-sectional | South Africa |  |
| Sibanda | 2017 | Effect of non-monetary incentives on uptake of couples’ counselling and testing among clients attending mobile HIV services in rural Zimbabwe: a cluster-randomised trial | | Randomized controlled | Zimbabwe |  |
| Sinha | 2017 | Integration and decentralisation of TB-HIV services increases HIV testing of TB cases in Rajasthan, India | | Quasi-experimental | India |  |
| Siril | 2017 | CLINICAL outcomes and loss to follow‑up among people living with HIV participating in the NAMWEZA intervention in Dar es Salaam, Tanzania: a prospective cohort study | | Cohort | Tanzania |  |
| Stevens | 2017 | Multidisciplinary Point-of-Care Testing in South African Primary Health Care Clinics Accelerates HIV ART Initiation but does not alter retention in care | | Randomized controlled | South Africa |  |
| Vanobberghen | 2017 | A decade of HIV care in rural Tanzania: Trends in clinical outcomes and impact of clinic optimisation in an open, prospective cohort | | Cohort | Tanzania |  |
| Weinhardt | 2017 | Mixed-Method Quasi-Experimental Study of Outcomes of a Large-Scale Multilevel Economic and Food Security Intervention on HIV Vulnerability in Rural Malawi | | Mixed methods | Malawi |  |
| Wu | 2017 | Testing and Linkage to HIV care in China: a cluster-randomized trial | | Randomized controlled | People's Republic of China |  |
| Zanoni | 2017 | Higher retention and viral suppression with adolescent-focused HIV clinic in South Africa | | Cohort | South Africa |  |
| Agaba | 2018 | Retention in Differentiated Care: Multiple Measures Analysis for a Decentralized HIV Care and Treatment Program in North Central Nigeria | | Cohort | Nigeria |  |
| Agala | 2018 | Organizational network strengthening effects on antiretroviral therapy initiation and adherence | | Cohort | Ethiopia |  |
| Akama | 2018 | Impact of a Rapid Results Initiative Approach on Improving Male Partner Involvement in Prevention of Mother to Child Transmission of HIV in Western Kenya | | Quasi-experimental | Kenya |  |
| Amzel | 2018 | Community-Based Interventions to Reach 95-95-95 for Children and Adolescents: An Exploratory Programmatic Review From Lesotho | | Program evaluation | Lesotho |  |
| Ayieko | 2018 | Effect of a Patient-Centered Phone Call by a Clinical Officer at Time of HIV Testing on Linkage to Care in Rural Kenya | | Randomized controlled | Kenya |  |
| Bacha | 2018 | The Standardized Pediatric Expedited Encounters for ART Drugs Initiative (SPEEDI): description and evaluation of an innovative pediatric, adolescent, and young adult antiretroviral service delivery model in Tanzania | | Cohort | Tanzania |  |
| Bekolo | 2018 | Feasibility of integrating HIV testing into local youth development programmes in Cameroon | | Program evaluation | Cameroon |  |
| Berheto | 2018 | Body and mind: retention in antiretroviral treatment care is improved by mental health training of care providers in Ethiopia | | Cohort | Ethiopia |  |
| Bermudez | 2018 | Does Economic Strengthening Improve Viral Suppression Among Adolescents Living with HIV? Results From a Cluster Randomized Trial in Uganda | | Randomized controlled | Uganda |  |
| Bhattacharjee | 2018 | Micro-planning at scale with key populations in Kenya: Optimising peer educator ratios for programme outreach and HIV/STI service utilisation | | Cohort | Kenya |  |
| Boeke | 2018 | Results from a proactive follow-up intervention to improve linkage and retention among people living with HIV in Uganda: a pre-/post- study | | Cohort | Uganda |  |
| Chamie | 2018 | Comparative effectiveness of novel non-monetary incentives to promote HIV testing: a randomized trial | | Randomized controlled | Uganda |  |
| Cho | 2018 | School Support as Structural HIV Prevention for Adolescent Orphans in Western Kenya | | Randomized controlled | Kenya |  |
| Courtenay-Quirk | 2018 | Increasing partner HIV testing and linkage to care in TB settings: findings from an implementation study in Pwani, Tanzania | | Randomized controlled | Tanzania |  |
| Cowan | 2018 | Targeted combination prevention to support female sex workers in Zimbabwe accessing and adhering to antiretrovirals for treatment and prevention of HIV (SAPPH-IRe): a cluster-randomised trial | | Randomized controlled | Zimbabwe | 10.1093/heapol/czz037 |
| de Jager | 2018 | Patient satisfaction and treatment adherence of stable human immunodeficiency virus-positive patients in in antiretroviral adherence clubs and clinics | | Cross-sectional | South Africa |  |
| Fatti | 2018 | Effectiveness of community-based support for pregnant women living with HIV: a cohort study in South Africa | | Cohort | South Africa |  |
| Fatti | 2018 | The effectiveness and cost-effectiveness of community-based support for adolescents receiving antiretroviral treatment: an operational research study in South Africa | | Cohort | South Africa |  |
| Fernandez-Luis | 2018 | Reengagement of HIV-infected children lost to follow-up after active mobile phone tracing in a rural area of Mozambique | | Cohort | Mozambique |  |
| Finocchario-Kessler | 2018 | Evaluation of the HIV Infant Tracking System (HITSystem) to optimise quality and efficiency of early infant diagnosis: a cluster-randomised trial in Kenya | | Randomized controlled | Kenya |  |
| Fox | 2018 | Effectiveness of interventions for unstable patients on antiretroviral therapy in South Africa: results of a cluster-randomised evaluation. | | Randomized controlled | South Africa |  |
| Geldsetzer | 2018 | Community delivery of antiretroviral drugs: A non-inferiority cluster-randomized pragmatic trial in Dar es Salaam, Tanzania | | Randomized controlled | Tanzania |  |
| Gichangi | 2018 | Impact of HIV Self-Test Distribution to Male Partners ofANC Clients: Results of a Randomized Controlled Trial in Kenya | | Randomized controlled | Kenya |  |
| Graves | 2018 | Impact of a Family Clinic Day intervention on paediatric and adolescent appointment adherence and retention in antiretroviral therapy: A cluster randomized controlled trial in Uganda | | Randomized controlled | Uganda |  |
| Green | 2018 | From conventional to disruptive: upturning the HIV testing status quo among men who have sex with men in Vietnam | | Cohort | Vietnam |  |
| He | 2018 | An innovative HIV testing service using the internet: Anonymous urine delivery testing service at drugstores in Beijing, China | | Cohort | People's Republic of China |  |
| Holden | 2018 | Building resilience to adverse childhood experiences: An assessment of the effects of the Stepping Stones with Children training programme on Tanzanian children affected by HIV and their caregivers | | Case-control | Tanzania |  |
| Jani | 2018 | Effect of point-of-care early infant diagnosis on antiretroviral therapy initiation and retention of patients | | Randomized controlled | Mozambique |  |
| Jani | 2018 | Effect of point-of-care early infant diagnosis on antiretroviral therapy initiation and retention of patients: a cluster-randomised trial | | Randomized controlled | Mozambique |  |
| Kalichman | 2018 | Stigma management intervention to improve antiretroviral therapy adherence: Phase-I test of concept trial, Cape Town South Africa | | Randomized controlled | South Africa |  |
| Kose | 2018 | Impact of a Comprehensive Adolescent-Focused Case Finding Intervention on Uptake of HIV Testing and Linkage to Care Among Adolescents in Western Kenya | | Quasi-experimental | Kenya |  |
| Kranzer | 2018 | Economic incentives for HIV testing by adolescents in Zimbabwe: a randomised controlled trial | | Randomized controlled | Zimbabwe |  |
| Kufa | 2018 | An intervention to optimise the delivery of integrated tuberculosis and HIV services at primary care clinics: results of the MERGE cluster randomised trial | | Randomized controlled | South Africa |  |
| Labhardt | 2018 | Effect of Offering Same-Day ART vs Usual Health Facility Referral During Home-Based HIV Testing on Linkage to Care and Viral Suppression Among Adults With HIV in Lesotho: The CASCADE Randomized Clinical Trial | | Randomized controlled | Lesotho | 10.1186/s12889-016-2972-6, 10.1186/s12889-019-7784-z, 10.1093/cid/ciz1126 |
| Lafort | 2018 | Effect of a ‘diagonal’ intervention on uptake of HIV and reproductive health services by female sex workers in three sub‐Saharan African cities | | Quasi-experimental | Kenya, Mozambique, South Africa |  |
| Langwenya | 2018 | Same-day antiretroviral therapy (ART) initiation in pregnancy is not associated with viral suppression or engagement in care: A cohort study | | Cohort | South Africa |  |
| Lelutiu-Weinberger | 2018 | An mHealth Intervention to Improve Young Gay and Bisexual Men’s Sexual, Behavioral, and Mental Health in a Structurally Stigmatizing National Context | | Cohort | Romania |  |
| Li | 2018 | An evaluation of impact of social support and care-giving on medication adherence of people living with HIV/AIDS | | Program evaluation | People's Republic of China |  |
| Lopez-Varela | 2018 | Continuum of HIV Care in Rural Mozambique: The Implications of HIV Testing Modality on Linkage and Retention | | Cohort | Mozambique |  |
| Loya-Montiel | 2018 | Making the Link: A Pilot Health Navigation Intervention to Improve Timely Linkage to Care for Men Who have Sex with Men and Transgender Women Recently Diagnosed with HIV in Guatemala City | | Mixed methods | Guatemala |  |
| Maughan-Brown | 2018 | A Conditional Economic Incentive Fails to Improve Linkage to Care and Antiretroviral Therapy Initiation Among HIV-Positive Adults in Cape Town, South Africa | | Randomized controlled | South Africa |  |
| Mboup | 2018 | Early antiretroviral therapy and daily pre-exposure prophylaxis for HIV prevention among female sex workers in Cotonou, Benin: a prospective observational demonstration study | | Cohort | Benin |  |
| McLaughlin | 2018 | Community-Based Accompaniment with Supervised Antiretrovirals for HIV-Positive Adults in Peru: A Cluster- Randomized Trial | | Randomized controlled | Peru |  |
| Mehta | 2018 | ‘M-TRACK’ (mobile phone reminders and electronic tracking tool) cuts the risk of pre-treatment loss to follow-up by 80% among people living with HIV under programme settings: a mixed-methods study from Gujarat, India | | Mixed methods | India |  |
| Miller | 2018 | Randomized Controlled Pilot Study of Antiretrovirals and a Behavioral Intervention for Persons With Acute HIV Infection: Opportunity for Interrupting Transmission | | Randomized controlled | Malawi |  |
| Miller | 2018 | A scalable, integrated intervention to engage people who inject drugs in HIV care and medication-assisted treatment (HPTN 074): a randomised, controlled phase 3 feasibility and efficacy study | | Randomized controlled | Indonesia, Ukraine, Vietnam |  |
| Mills | 2018 | Unconditional cash transfers for clinical and economic outcomes among HIV-affected Ugandan households | | Randomized controlled | Uganda |  |
| Mody | 2018 | Improved Retention With 6-Month Clinic Return Intervals for Stable Human Immunodeficiency Virus-Infected Patients in Zambia | | Cohort | Zambia |  |
| Mulubwa | 2018 | Community based distribution of oral HIV self-testing kits in Zambia: a cluster-randomised trial nested in four HPTN 071 (PopART) intervention communities | | Randomized controlled | Zambia | 10.1097/QAI.0000000000002344 |
| Musarandega | 2018 | Scaling up Pediatric HIV Testing by Incorporating Provider-Initiated HIV Testing Into all Child Health Services in Hurungwe District, Zimbabwe | | Quasi-experimental | Zimbabwe |  |
| Myer | 2018 | Integration of postpartum healthcare services for HIV-infected women and their infants in South Africa: A randomised controlled trial | | Randomized controlled | South Africa | 10.1186/s12913-020-05470-5 |
| Nsirim | 2018 | Effectiveness of provider-initiated testing and counseling in increasing HIV testing and counselling utilization and HIV detection rates in Ebonyi State, South-Eastern Nigeria | | Cohort | Nigeria |  |
| Odeny | 2018 | Participation in a clinical trial of a text messaging intervention is associated with increased infant HIV testing: A parallel-cohort randomized controlled trial | | Randomized controlled | Kenya |  |
| Oladele | 2018 | Bridging the HIV treatment gap in Nigeria: examining community antiretroviral treatment models | | Randomized controlled | Nigeria |  |
| Phanuphak | 2018 | Princess PrEP program: the first key population-led model to deliver pre-exposure prophylaxis to key populations by key populations in Thailand | | Program evaluation | Thailand |  |
| Pokhrel | 2018 | Investigating the impact of a community home-based care on mental health and anti-retroviral therapy adherence in people living with HIV in Nepal: a community intervention study | | Cohort | Nepal |  |
| Prochazka | 2018 | Patient-nominated supporters as facilitators for engagement in HIV care in a referral hospital in Peru: A retrospective cohort study | | Cohort | Peru |  |
| Rentsch | 2018 | Linkage to care and antiretroviral therapy initiation by testing modality among individuals newly diagnosed with HIV in Tanzania, 2014–2017 | | Cohort | Tanzania |  |
| Shah | 2018 | A Pilot Study of Peer Navigators to Promote Uptake of HIV Testing, Care and Treatment Among Street-Connected Children and Youth in Eldoret, Kenya | | Cohort | Kenya |  |
| Shamu | 2018 | Social franchising of community‐based HIV testing and linkage to HIV care and treatment services: an evaluation of a pilot study in Tshwane, South Africa | | Case-control | South Africa |  |
| Sindelar | 2018 | Beyond the facility: An evaluation of seven community-based pediatric HIV testing strategies and linkage to care outcomes in a high prevalence, resource-limited setting | | Cohort | South Africa |  |
| Tang | 2018 | Crowdsourcing to expand HIV testing among men who have sex with men in China: A closed cohort stepped wedge cluster randomized controlled trial | | Randomized controlled | People's Republic of China |  |
| Technau | 2018 | 12-month outcomes of HIV-infected infants identified at birth at one maternity site in Johannesburg, South Africa: an observational cohort study | | Cohort | South Africa |  |
| Tun | 2018 | Uptake of HIV self‐testing and linkage to treatment among men who have sex with men (MSM) in Nigeria: A pilot programme using key opinion leaders to reach MSM | | Cohort | Nigeria |  |
| vanderKop | 2018 | Effect of an interactive text-messaging service on patient retention during the first year of HIV care in Kenya (WelTel Retain): an open-label, randomised parallel-group study | | Randomized controlled | Kenya |  |
| Wesevich | 2018 | Role of male partner involvement in ART retention and adherence in Malawi’s Option B+ program | | Cohort | Malawi |  |
| Wroe | 2018 | Delivering comprehensive HIV services across the HIV care continuum: a comparative analysis of survival and progress towards 90-90-90 in rural Malawi | | Program evaluation | Malawi |  |
| Xia | 2018 | Feasibility of an internet-based HIV testing service: anonymous urine collection from men who have sex with men | | Cohort | People's Republic of China |  |
| Ye | 2018 | Promoting access equity and improving health care for women, children and people living with HIV/AIDS in Burkina Faso through mHealth | | Cohort | Burkina Faso |  |
| Yumo | 2018 | Active case finding: comparison of the acceptability, feasibility and effectiveness of targeted versus blanket provider-initiated-testing and counseling of HIV among children and adolescents in Cameroon | | Case-control | Cameroon |  |
| Adinan | 2019 | Feasibility of home-based HIV counselling and testing and linking to HIV services among women delivering at home in Geita, Tanzania: a household longitudinal survey | | Cross-sectional | Tanzania |  |
| Ahmadi | 2019 | The effectiveness of peer education interventions on HIV- and HBV-preventive behaviours in women with substance-related disorders: a cluster randomised control trial | | Randomized controlled | Iran |  |
| Alhaj | 2019 | Retention on antiretroviral therapy during Universal Test andTreat implementation in Zomba district, Malawi: a retrospectivecohort study | | Cohort | Malawi |  |
| Alhassan | 2019 | Impact of a bottom-up community engagement intervention on maternal and child health services utilization in Ghana: a cluster randomised trial | | Randomized controlled | Ghana |  |
| Alizadeh | 2019 | Identifying and Reengaging Patients Lost to Follow-Up in Rural Africa: The “Horizontal” Hospital-Based Approach in Uganda | | Cohort | Uganda |  |
| Azuogu | 2019 | Effect of multiple intervention models on uptake of HIV testing services and sexual behaviour among residents of military cantonments in southeast Nigeria | | Quasi-experimental | Nigeria |  |
| Baisley | 2019 | Findings from home-based HIV testing and facilitated linkage after scale-up of test and treat in rural South Africa: young people still missing | | Cohort | South Africa |  |
| Barker | 2019 | In-Clinic Adolescent Peer Group Support for Engagement in Sub-Saharan Africa: A Feasibility and Acceptability Trial | | Cohort | Ghana |  |
| Birungi | 2019 | Lack of effectiveness of adherence counselling in reversing virological failure among patients on long‐term antiretroviral therapy in rural Uganda | | Cohort | Uganda |  |
| Bochner | 2019 | Strengthening provider-initiated testing and counselling in Zimbabwe by deploying supplemental providers: a time series analysis | | Quasi-experimental | Zimbabwe |  |
| Bock | 2019 | Retention in care and factors critical for effectively implementing antiretroviral adherence clubs in a rural district in South Africa | | Cohort | South Africa |  |
| Boni | 2019 | An Internet-Based HIV Self-Testing Program to Increase HIV Testing Uptake Among Men Who Have Sex With Men in Brazil: Descriptive Cross-Sectional Analysis | | Cross-sectional | Brazil |  |
| Bui | 2019 | Couples HIV testing and immediate antiretroviral therapy for serodiscordant HIV-positive partners: Translating evidence into programme in Vietnam | | Cohort | Vietnam |  |
| Bvochora | 2019 | Enhanced Adherence Counselling and Viral Load Suppression in HIV Seropositive Patients With an Initial High Viral Load in Harare, Zimbabwe: Operational Issues | | Cohort | Zimbabwe |  |
| Cao | 2019 | Recalling, sharing and participating in a social media intervention promoting HIV testing: A longitudinal analysis of HIV testing among MSM in China | | Quasi-experimental | People's Republic of China |  |
| Cham | 2019 | Methods, outcomes, and costs of a 2.5 year comprehensive facility-and community-based HIV testing intervention in Bukoba Municipal Council, Tanzania, 2014-2017 | | Cohort | Tanzania | 10.1016/S2352-3018(20)30199-5 |
| Darbes | 2019 | Results of a couples-based randomized controlled trial aimed to increase testing for HIV | | Randomized controlled | South Africa |  |
| Dougherty | 2019 | Reaching the First 90: Improving Inpatient Pediatric Provider-Initiated HIV Testing and Counseling Using a Quality Improvement Collaborative Strategy in Tanzania | | Quasi-experimental | Tanzania |  |
| Duflo | 2019 | HIV prevention among youth: A randomized controlled trial of voluntary counseling and testing for HIV and male condom distribution in rural Kenya | | Randomized controlled | Kenya |  |
| Dulli | 2019 | Addressing broader reproductive health needs of female sex workers through integrated family planning/ HIV prevention services: A non-randomized trial of a health-services intervention designed to improve uptake of family planning services in Kenya | | Quasi-experimental | Kenya |  |
| Fayorsey | 2019 | Effectiveness of a Lay Counselor–Led Combination Intervention for Retention of Mothers and Infants in HIV Care: A Randomized Trial in Kenya | | Randomized controlled | Kenya |  |
| Fox | 2019 | Adherence clubs and decentralized medication delivery to support patient retention and sustained viral suppression in care: Results from a cluster-randomized evaluation of differentiated ART delivery models in South Africa | | Randomized controlled | South Africa |  |
| Gbadamosi | 2019 | Targeted HIV testing for male partners of HIV-positive pregnant women in a high prevalence setting in Nigeria | | Cohort | Nigeria | 10.1097/qai.0000000000001051 |
| Gourlay | 2019 | Awareness and uptake of layered HIV prevention programming for young women: analysis of population-based surveys in three DREAMS settings in Kenya and South Africa | | Cohort | Kenya, South Africa |  |
| Hacking | 2019 | Peer Mentorship via Mobile Phones for Newly Diagnosed HIV-Positive Youths in Clinic Care in Khayelitsha, South Africa: Mixed Methods Study | | Case-control | South Africa |  |
| Hanrahan | 2019 | The impact of community- versus clinic-based adherence clubs on loss from care and viral suppression for antiretroviral therapy patients: Findings from a pragmatic randomized controlled trial in South Africa | | Randomized controlled | South Africa |  |
| Havlir | 2019 | HIV Testing and Treatment with the Use of a Community Health Approach in Rural Africa | | Randomized controlled | Kenya, Uganda | 10.1016/S2352-3018(15)00251-9, 10.1097/qai.0000000000001141, 10.7448/ias.20.5.21673, 10.1002/jia2.25037, 10.1097/QAI.0000000000001939, 10.1097/inf.0000000000002142, 10.1002/jia2.25148, 10.1097/QAD.0000000000001958, 10.1093/cid/ciaa1782, 10.1016/S2352-3018(19)30433-3, 10.1371/journal.pmed.1003492 |
| Hayes | 2019 | Effect of Universal Testing and Treatment on HIV Incidence — HPTN 071 (PopART) | | Randomized controlled | South Africa, Zambia | 10.1371/journal.pmed.1002292, 10.1371/journal.pone.0197904, 10.1007/s10461-018-2335-7, 10.1093/cid/ciz214, 10.1371/journal.pmed.1003067, 10.1016/j.jadohealth.2020.07.029 |
| Heffron | 2019 | Implementation of a comprehensive safer conception intervention for HIV‐serodiscordant couples in Kenya: uptake, use and effectiveness | | Cohort | Kenya |  |
| Homsy | 2019 | Primary HIV prevention in pregnant and lactating Ugandan women: A randomized trial | | Randomized controlled | Uganda |  |
| Htet | 2019 | Early Success With Retention in Care Among People Living With HIV at Decentralized ART Satellite Sites in Yangon, Myanmar, 2015–2016 | | Cohort | Myanmar |  |
| Jubilee | 2019 | HIV index testing to improve HIV positivity rate and linkage to care and treatment of sexual partners, adolescents and children of PLHIV in Lesotho | | Cohort | Lesotho |  |
| Katbi | 2019 | Effect of community treatment initiative on antiretroviral therapy uptake among linkage-resistant people living with HIV in Northern Nigeria | | Program evaluation | Nigeria |  |
| Kelvin | 2019 | Announcing the availability of oral HIV selftest kits via text message to increase HIV testing among hard-to-reach truckers in Kenya: a randomized controlled trial | | Randomized controlled | Kenya |  |
| Kerrigan | 2019 | Project Shikamana: community empowerment-based combination HIV prevention significantly impacts HIV incidence and care continuum outcomes among female sex workers in Iringa, Tanzania | | Randomized controlled | Tanzania |  |
| Kuznetsova | 2019 | Linking intravenous drug users to treatment through non-governmental organizations in Ukraine: how well is it working? | | Cohort | Ukraine |  |
| Le Roux | 2019 | Antiretroviral Therapy Program in Rural South Africa | | Cohort | South Africa |  |
| Liu | 2019 | Conditional cash transfers to prevent mother-to-child transmission in low facility-delivery settings: evidence from a randomised controlled trial in Nigeria | | Randomized controlled | Nigeria |  |
| Makhema | 2019 | Universal Testing, Expanded Treatment, and Incidence of HIV Infection in Botswana | | Randomized controlled | Botswana | 10.1016/S2352-3018(20)30187-9, 10.1371/journal.pone.0255227 |
| Mark | 2019 | Male Partner Linkage to Clinic-Based Services for Sexually Transmitted Infections and Human Immunodeficiency Virus Services Following Couple Home-Based Education and Testing | | Randomized controlled | Kenya |  |
| Masereka | 2019 | Increasing retention of HIV positive pregnant and breastfeeding mothers on option-b plus by upgrading and providing full time HIV services at a lower health facility in rural Uganda | | Cross-sectional | Uganda |  |
| Mashaphu | 2019 | Effectiveness of an HIV-risk reduction intervention to reduce HIV transmission among serodiscordant couples in Durban, South Africa. A randomized controlled trial | | Randomized controlled | South Africa |  |
| Masyuko | 2019 | Index participant characteristics and HIV assisted partner services efficacy in Kenya: results of a cluster randomized trial | | Randomized controlled | Kenya |  |
| Mebrahtu | 2019 | Effects of parenting classes and economic strengthening for caregivers on the cognition of HIV-exposed infants: a pragmatic cluster randomised controlled trial in rural Zimbabwe | | Randomized controlled | Zimbabwe |  |
| Moucheraud | 2019 | Can Self-Management Improve HIV Treatment Engagement, Adherence, and Retention? A Mixed Methods Evaluation in Tanzania and Uganda | | Quasi-experimental | Tanzania, Uganda |  |
| Muchabaiwa | 2019 | Impact of the adolescent and youth sexual and reproductive health strategy on service utilisation and health outcomes in Zimbabwe | | Quasi-experimental | Zimbabwe |  |
| Odeny | 2019 | Text messaging for maternal and infant retention in prevention of mother-to-child HIV transmission services: A pragmatic stepped-wedge cluster-randomized trial in Kenya | | Randomized controlled | Kenya |  |
| Pascoe | 2019 | Differentiated HIV care in South Africa: the effect of fast‐track treatment initiation counselling on ART initiation and viral suppression as partial results of an impact evaluation on the impact of a package of services to improve HIV treatment adherence | | Randomized controlled | South Africa |  |
| Perez-Patrigeon | 2019 | An interdisciplinary approach for immediate ART initiation in patients with acute HIV infection in Mexico City | | Cohort | Mexico |  |
| Phiri | 2019 | Impact of the Umoyo mother-infant pair model on HIV-positive mothers’ social support, perceived stigma and 12-month retention of their HIV-exposed infants in PMTCT care: evidence from a cluster randomized controlled trial in Zambia | | Randomized controlled | Zambia |  |
| Ramlagan | 2019 | Self-Reported Long-Term Antiretroviral Adherence: A Longitudinal Study Among HIV Infected Pregnant Women in Mpumalanga, South Africa | | Randomized controlled | South Africa |  |
| Reif | 2019 | “FANMI”: A Promising Differentiated Model of HIV Care for Adolescents in Haiti | | Cohort | Haiti |  |
| Reza-Paul | 2019 | Sex Worker Community-led Interventions Interrupt Sexually Transmitted Infection/Human Immunodeficiency Virus Transmission and Improve Human Immunodeficiency Virus Cascade Outcomes: A Program Review from South India | | Cohort | India |  |
| Rosen | 2019 | Simplified clinical algorithm for identifying patients eligible for same-day HIV treatment initiation (SLATE): Results from an individually randomized trial in South Africa and Kenya | | Randomized controlled | Kenya, South Africa |  |
| Ross | 2019 | Early outcomes after implementation of treat all in Rwanda: an interrupted time series study | | Quasi-experimental | Rwanda |  |
| Sabin | 2019 | Effectiveness of community outreach HIV prevention programs in Vietnam: a mixed methods evaluation | | Cross-sectional | Vietnam |  |
| Sanders | 2019 | Point-of-care HIV RNA testing and immediate antiretroviral therapy initiation in young adults seeking out-patient care in Kenya | | Cohort | Kenya |  |
| Sarna | 2019 | Cell Phone Counseling Improves Retention of Mothers With HIV Infection in Care and Infant HIV Testing in Kisumu, Kenya: A Randomized Controlled Study | | Randomized controlled | Kenya |  |
| Schwartz | 2019 | HIV incidence, pregnancy and implementation outcomes from the Sakh’umndeni Safer Conception Project in South Africa: a prospective cohort study | | Cohort | South Africa |  |
| Simon | 2019 | The Tingathe Surge: a multi-strategy approach to accelerate HIV case finding in Malawi | | Program evaluation | Malawi |  |
| SmithFawzi | 2019 | Agents of change among people living with HIV and their social networks: stepped-wedge randomised controlled trial of the NAMWEZA intervention in Dar es Salaam, Tanzania | | Randomized controlled | Tanzania |  |
| Tapera | 2019 | Effects of a Peer-Led Intervention on HIV Care Continuum Outcomes Among Contacts of Children, Adolescents, and Young Adults Living With HIV in Zimbabwe | | Cohort | Zimbabwe |  |
| Thirumurthy | 2019 | Financial incentives for achieving and maintaining viral suppression among HIV-positive adults in Uganda: a randomised controlled trial | | Randomized controlled | Uganda |  |
| Truong | 2019 | Implementation of a Community-Based Hybrid HIV Testing Services Program as a Strategy to Saturate Testing Coverage in Western Kenya | | Cohort | Kenya |  |
| Tun | 2019 | Community-Based Antiretroviral Therapy (ART) Delivery for Female Sex Workers in Tanzania: 6-Month ART Initiation and Adherence | | Cohort | Tanzania |  |
| Venter | 2019 | Improving Linkage to and Retention in Care in Newly Diagnosed HIV-Positive Patients Using Smartphones in South Africa: Randomized Controlled Trial | | Randomized controlled | South Africa |  |
| Vrana-Diaz | 2019 | Relationship Gender Equality and Couples' Uptake of Oral Human Immunodeficiency Virus Self-Testing Kits Delivered by Pregnant Women in Kenya | | Randomized controlled | Kenya |  |
| Wanless | 2019 | A comparison of adherence to antiretroviral therapy amongst adolescent patients of the Baylor International Pediatric AIDS Initiative Children’s Centers of Excellence before and after attendance at recreational therapy camp | | Cohort | eSwatini, Lesotho, Malawi, Uganda |  |
| Willis | 2019 | Effectiveness of community adolescent treatment supporters (CATS) interventions in improving linkage and retention in care, adherence to ART and psychosocial well-being: a randomised trial among adolescents living with HIV in rural Zimbabwe | | Randomized controlled | Zimbabwe |  |
| Abubakari | 2020 | Implementation and evaluation of a culturally grounded group-based HIV prevention programme for men who have sex with men in Ghana | | Mixed methods | Ghana |  |
| Albaugh | 2020 | Proportion and predictors of adult TB contacts accepting HIV testing during an active TB case finding intervention in South Africa | | Randomized controlled | South Africa |  |
| Amirkhanian | 2020 | Mobilizing individual social capital resources for HIV care support: results of a pilot intervention in St. Petersburg, Russia | | Quasi-experimental | Russia |  |
| Amstutz | 2020 | Home-based oral self-testing for absent and declining individuals during a door-to-door HIV testing campaign in rural Lesotho (HOSENG): a cluster-randomised trial | | Randomized controlled | Lesotho | 10.1002/jia2.25563 |
| Asieba | 2020 | Antiretroviral therapy in community pharmacies - Implementation and outcomes of a differentiated drug delivery model in Nigeria | | Cohort | Nigeria |  |
| Barnabas | 2020 | Lottery incentives have short-term impact on ART initiation among men: results from a randomized pilot study | | Randomized controlled | South Africa |  |
| Barnabas | 2020 | Community-based antiretroviral therapy versus standard clinic-based services for HIV in South Africa and Uganda (DOART): a randomised trial | | Randomized controlled | South Africa, Uganda |  |
| Cassidy | 2020 | Twenty‐four‐month outcomes from a cluster‐randomized controlled trial of extending antiretroviral therapy refills in ART adherence clubs | | Randomized controlled | South Africa |  |
| Chamie | 2020 | A pilot randomized trial of incentive strategies to promote HIV retesting in rural Uganda | | Randomized controlled | Uganda |  |
| Chatha | 2020 | Pharmacist-led counselling intervention to improve antiretroviral drug adherence in Pakistan: a randomized controlled trial | | Randomized controlled | Pakistan |  |
| Dawson-Rose | 2020 | Effects of a peer educator program for HIV status disclosure and health system strengthening: Findings from a clinic-based disclosure support program in Mozambique | | Cohort | Mozambique |  |
| Denison | 2020 | Project YES! Youth Engaging for Success: A randomized controlled trial assessing the impact of a clinic-based peer mentoring program on viral suppression, adherence and internalized stigma among HIV-positive youth (15-24 years) in Ndola, Zambia | | Randomized controlled | Zambia |  |
| Diallo | 2020 | HIV treatment response among female sex workers participating in a treatment as prevention demonstration project in Cotonou, Benin | | Cohort | Benin |  |
| Dovel | 2020 | Effect of facility-based HIV self-testing on uptake of testing among outpatients in Malawi: a cluster-randomised trial | | Randomized controlled | Malawi | 10.1002/jia2.25612 |
| Dow | 2020 | A group-based mental health intervention for young people living with HIV in Tanzania: results of a pilot individually randomized group treatment trial | | Randomized controlled | Tanzania |  |
| Drain | 2020 | Point-of-care HIV viral load testing combined with task shifting to improve treatment outcomes (STREAM): findings from an open-label, non-inferiority, randomised controlled trial | | Randomized controlled | South Africa |  |
| Drake | 2020 | The Utility of SMS to Report Male Partner HIV Self-testing Outcomes Among Women Seeking Reproductive Health Services in Kenya: Cohort Study | | Cohort | Kenya |  |
| Dulli | 2020 | A Social Media-Based Support Group for Youth Living With HIV in Nigeria (SMART Connections): Randomized Controlled Trial | | Randomized controlled | Nigeria |  |
| DzivaChikwari | 2020 | Comparison of index-linked HIV testing for children and adolescents in health facility and community settings in Zimbabwe: finding from the interventional B-GAP study | | Program evaluation | Zimbabwe |  |
| Ekstrand | 2020 | A Behavioral Adherence Intervention Improves Rates of Viral Suppression Among Adherence‑Challenged People Living with HIV | | Randomized controlled | India |  |
| Fahey | 2020 | Financial incentives to promote retention in care and viral suppression in adults with HIV initiating antiretroviral therapy in Tanzania: a three-arm randomised controlled trial | | Randomized controlled | Tanzania |  |
| Fatti | 2020 | Outcomes of Three- Versus Six-Monthly Dispensing of Antiretroviral Treatment (ART) for Stable HIV Patients in Community ART Refill Groups: A Cluster-Randomized Trial in Zimbabwe | | Randomized controlled | Zimbabwe |  |
| Fiorentino | 2020 | Early ART Initiation Improves HIV Status Disclosure and Social Support in People Living with HIV, Linked to Care Within a Universal Test and Treat Program in Rural South Africa (ANRS 12249 TasP Trial) | | Randomized controlled | South Africa |  |
| Garg | 2020 | Mobile Health App for Self-Learning on HIV Prevention Knowledge and Services Among a Young Indonesian Key Population: Cohort Study | | Quasi-experimental | Indonesia |  |
| Graham | 2020 | A Randomized Controlled Trial of the Shikamana Intervention to Promote Antiretroviral Therapy Adherence Among Gay, Bisexual, and Other Men Who Have Sex with Men in Kenya: Feasibility, Acceptability, Safety and Initial Effect Size | | Randomized controlled | Kenya |  |
| Herce | 2020 | Universal test-and-treat in Zambian and South African correctional facilities: a multisite prospective cohort study | | Cohort | South Africa, Zambia |  |
| Hopkins | 2020 | Does peer-navigated linkage to care work? A cross-sectional study of active linkage to care within an integrated non-communicable disease-HIV testing centre for adults in Soweto, South Africa | | Cohort | South Africa |  |
| Kassa | 2020 | Improving inpatient provider-initiated HIV testing and counseling in Sierra Leone | | Cohort | Sierra Leone |  |
| Katz | 2020 | The Treatment Ambassador Program: A Highly Acceptable and Feasible Community‑Based Peer Intervention for South Africans Living with HIV Who Delay or Discontinue Antiretroviral Therapy | | Randomized controlled | South Africa |  |
| Kavanagh | 2020 | Planning prompts to promote uptake of HIV services among men: a randomised trial in rural Uganda | | Randomized controlled | Uganda |  |
| Kerschberger | 2020 | HIV programmatic outcomes following implementation of the ‘Treat-All’ policy in a public sector setting in Eswatini: a prospective cohort study | | Cohort | eSwatini |  |
| Khan | 2020 | Early access to antiretroviral therapy versus standard of care among HIV-positive participants in Eswatini in the public health sector: the MaxART stepped-wedge randomized controlled trial | | Randomized controlled | eSwatini |  |
| Khumalo | 2020 | The Cascade of Care From Routine Point-of-Care HIV Testing at Birth: Results From an 18-Months Pilot Program in Eswatini | | Program evaluation | eSwatini |  |
| Kingbo | 2020 | Partner Notification Approaches for Sex Partners and Children of Human Immunodeficiency Virus Index Cases in Côte d'Ivoire | | Cohort | Ivory Coast |  |
| Korte | 2020 | HIV Oral Self-Testing for Male Partners of Women Attending Antenatal Care in Central Uganda: Uptake of Testing and Linkage to Care in a Randomized Trial | | Randomized controlled | Uganda |  |
| Kroidl | 2020 | High turnaround times and low viral resuppression rates after reinforced adherence counselling following a confirmed virological failure diagnostic algorithm in HIV‐infected patients on first‐line antiretroviral therapy from Tanzania | | Cohort | Tanzania |  |
| Lasry | 2020 | Outcome of HIV Testing Among Family Members of Index Cases Across 36 Facilities in Abidjan, Côte d’Ivoire | | Cohort | Ivory Coast |  |
| Lin | 2020 | Using repeated home-based HIV testing services to reach and diagnose HIV infection among persons who have never tested for HIV, Cho´kwè health demographic surveillance system, Cho´kwè district, Mozambique, 2014– 2017 | | Cross-sectional | Mozambique |  |
| Lolekha | 2020 | Implementation of an active case management network to identify HIV-positive infants and accelerate the initiation of antiretroviral therapy, Thailand 2015 to 2018 | | Cohort | Thailand |  |
| MacCarthy | 2020 | A randomized controlled trial study of the acceptability, feasibility, and preliminary impact of SITA (SMS as an Incentive To Adhere): a mobile technology-based intervention informed by behavioral economics to improve ART adherence among youth in Uganda | | Randomized controlled | Uganda |  |
| Mackellar | 2020 | Overcoming Barriers to HIV Care: Findings from a Peer‑Delivered, Community‑Based, Linkage Case Management Program (CommLink), Eswatini, 2015–2018 | | Cohort | eSwatini |  |
| Malama | 2020 | A couple-focused, integrated unplanned pregnancy and HIV prevention program in urban and rural Zambia in South India | | Cohort | Zambia |  |
| Maman | 2020 | Results from a cluster-randomized trial to evaluate a microfinance and peer health leadership intervention to prevent HIV and intimate partner violence among social networks of Tanzanian men | | Randomized controlled | Tanzania |  |
| Mangombe | 2020 | Does peer education go beyond giving reproductive health information? Cohort study in Bulawayo and Mount Darwin, Zimbabwe | | Cohort | Zimbabwe |  |
| Maskew | 2020 | A clinical algorithm for same-day HIV treatment initiation in settings with high TB symptom prevalence in South Africa: The SLATE II individually randomized clinical trial | | Randomized controlled | South Africa |  |
| Matovu | 2020 | Feasibility and acceptability of a pilot, peer-led HIV self-testing intervention in a hyperendemic fishing community in rural Uganda | | Program evaluation | Uganda |  |
| Mavhu | 2020 | Effect of a differentiated service delivery model on virological failure in adolescents with HIV in Zimbabwe (Zvandiri): a cluster-randomised controlled trial | | Randomized controlled | Zimbabwe |  |
| Milford | 2020 | Ever and repeat HIV testing rates among male and female clients: Findings from a reproductive health services integration project in South Africa | | Cohort | South Africa |  |
| Moudachirou | 2020 | Retention and sustained viral suppression in HIV patients transferred to community refill centres in Kinshasa, DRC | | Cohort | Democratic Republic of the Congo |  |
| Mubiana-Mbewe | 2020 | Efect of Enhanced Adherence Package on Early ART Uptake Among HIV‑Positive Pregnant Women in Zambia: An Individual Randomized Controlled Trial | | Randomized controlled | Zambia |  |
| Mugo | 2020 | Home- and Clinic-Based Pediatric HIV Index Case Testing in Kenya: Uptake, HIV Prevalence, Linkage to Care, and Missed Opportunities | | Cohort | Kenya |  |
| Munyayi | 2020 | The effects of Teen Clubs on retention in HIV care among adolescents in Windhoek, Namibia | | Cohort | Namibia |  |
| Munyayi | 2020 | The Comparison of Teen Clubs vs. Standard Care on Treatment Outcomes for Adolescents on Antiretroviral Therapy in Windhoek, Namibia | | Cohort | Namibia |  |
| Mushamiri | 2020 | Optimizing PMTCT efforts by repeat HIV HIV testing during antenatal and perinatal care in resource-limited settings: A longitudinal assessment of HIV seroconversion | | Cohort | Kenya |  |
| Mwango | 2020 | Index and targeted community-based testing to optimize HIV case finding and ART linkage among men in Zambia | | Cohort | Zambia |  |
| Napierala | 2020 | Male partner testing and sexual behaviour following provision of multiple HIV self-tests to Kenyan women at higher risk of HIV infection in a cluster randomized trial | | Randomized controlled | Kenya |  |
| Naugle | 2020 | Addressing Uptake of HIV Testing and Linkage to Care Among Men in Côte d'Ivoire: An Evaluation of the Brothers for Life Program Implementation | | Program evaluation | Ivory Coast |  |
| Neduzhko | 2020 | Modified Antiretroviral Treatment Access Study (MARTAS): A Randomized Controlled Trial of the Efficacy of a Linkage-to-Care Intervention Among HIV-Positive Patients in Ukraine | | Randomized controlled | Ukraine |  |
| O'Laughlin | 2020 | A cohort study to assess a communication intervention to improve linkage to HIV care in Nakivale Refugee Settlement, Uganda | | Cohort | Uganda |  |
| Olawore | 2020 | Peer Recruitment Strategies for Female Sex Workers Not Engaged in HIV Prevention and Treatment Services in Côte d’Ivoire: Program Data Analysis | | Program evaluation | Ivory Coast |  |
| Pasipamire | 2020 | Implementation of community and facility-based HIV self-testing under routine conditions in southern Eswatini | | Cohort | eSwatini |  |
| Patel | 2020 | An Internet-Based, Peer-Delivered Messaging Intervention for HIV Testing and Condom Use Among Men Who Have Sex With Men in India (CHALO!): Pilot Randomized Comparative Trial | | Randomized controlled | India |  |
| Pettifor | 2020 | HIV self-testing among young women in rural South Africa: A randomized controlled trial comparing clinic-based HIV testing to the choice of either clinic testing or HIV self-testing with secondary distribution to peers and partners | | Randomized controlled | South Africa |  |
| Puttkammer | 2020 | An EMR-Based Alert with Brief Provider-led ART Adherence Counseling: Promising Results of the InfoPlus Adherence Pilot Study among Haitian Adults with HIV Initiating ART | | Mixed methods | Haiti |  |
| Roy | 2020 | Participation in adherence clubs and on-time drug pickup among HIV-infected adults in Zambia: A matched-pair cluster randomized trial | | Randomized controlled | Zambia |  |
| Sabin | 2020 | Retention in HIV Care Among HIV‑Seropositive Pregnant and Postpartum Women in Uganda: Results of a Randomized Controlled Trial | | Randomized controlled | Uganda |  |
| Sacks | 2020 | Impact of Routine Point-of-Care Versus Laboratory Testing for Early Infant Diagnosis of HIV: Results From a Multicountry Stepped-Wedge Cluster-Randomized Controlled Trial | | Randomized controlled | Kenya, Zimbabwe |  |
| Salvadori | 2020 | Appointment reminders to increase uptake of HIV retesting by at-risk individuals: a randomized controlled study in Thailand | | Randomized controlled | Thailand |  |
| Shapiro | 2020 | An implementation study of oral and blood-based HIV self-testing and linkage to care among men in rural and peri-urban KwaZulu-Natal, South Africa | | Program evaluation | South Africa |  |
| Suryavanshi | 2020 | A mobile health-facilitated behavioural intervention forcommunity health workers improves exclusive breastfeeding andearly infant HIV diagnosis in India: a cluster randomized trial | | Randomized controlled | India |  |
| Tanser | 2020 | Home-Based Intervention to Test and Start (HITS): a community-randomized controlled trial to increase HIV testing uptake among men in rural South Africa | | Randomized controlled | South Africa |  |
| Teasdale | 2020 | Birth Testing for Infant HIV Diagnosis in Eswatini: Implementation Experience and Uptake Among Women Living With HIV in Manzini Region | | Cohort | eSwatini |  |
| Truong | 2020 | Community-based HIV testing services in an urban setting in western Kenya: a programme implementation study | | Cross-sectional | Kenya |  |
| Tukei | 2020 | Twelve-Month Outcomes of Community-Based Differentiated Models of Multimonth Dispensing of ART Among Stable HIV-Infected Adults in Lesotho: A Cluster-Randomized Noninferiority Trial | | Randomized controlled | Lesotho |  |
| Tun | 2020 | Integration of HIV services with primary care in Yangon, Myanmar: a retrospective cohort analysis | | Cohort | Myanmar |  |
| Wanga | 2020 | Uptake and impact of facility-based HIV self-testing on PrEP delivery: a pilot study among young women in Kisumu, Kenya | | Cohort | Kenya |  |
| Xiao | 2020 | Sexual network distribution of HIV self-testing kits: Findings from the process evaluation of an intervention for men who have sex with men in China | | Randomized controlled | People's Republic of China |  |
| Zhang | 2020 | Impact of providing free HIV self-testing kits on frequency of testing among men who have sex with men and their sexual partners in China: A randomized controlled trial | | Randomized controlled | People's Republic of China |  |
| Abiodun | 2021 | A Single-Blind, Parallel Design RCT to Assess the Effectiveness of SMS Reminders in Improving ART Adherence Among Adolescents Living with HIV (STARTA Trial) | | Randomized controlled | Nigeria |  |
| Ayer | 2021 | Nurse‑Led Mobile Phone Voice Call Reminder and On‑Time Antiretroviral Pills Pick‑Up in Nepal: A Randomized Controlled Trial | | Randomized controlled | Nepal |  |
| Bachanas | 2021 | Finding, treating and retaining persons with HIV in a high HIV prevalence and high treatment coverage country: Results from the Botswana Combination Prevention Project | | Randomized controlled | Botswana |  |
| Boeke | 2021 | Universal test and treat in relation to HIV disease progression: results from a stepped-wedge trial in Eswatini | | Randomized controlled | eSwatini |  |
| Chamie | 2021 | Financial incentives and deposit contracts to promote HIV retesting in Uganda: A randomized trial | | Randomized controlled | Uganda |  |
| Chang | 2021 | Novel community health worker strategy for HIV service engagement in a hyperendemic community in Rakai, Uganda: A pragmatic, cluster-randomized trial | | Randomized controlled | Uganda |  |
| Chen | 2021 | A randomized controlled trial evaluating combination detection of HIV in Malawian sexually transmitted infections clinics | | Randomized controlled | Malawi |  |
| Cheng | 2021 | Efectiveness of HIV Self‑testing on Regular HIV Testing Among Ever‑Tested Men Who Have Sex Men in China: A Pragmatic Randomized Controlled Trial | | Randomized controlled | People's Republic of China |  |
| Choko | 2021 | Partner-delivered HIV self-test kits with and without financial incentives in antenatal care and index patients with HIV in Malawi: a three-arm, cluster-randomised controlled trial | | Randomized controlled | Malawi |  |
| Chory | 2021 | A Pilot Study of a Mobile Intervention to Support Mental Health and Adherence Among Adolescents Living with HIV in Western Kenya | | Mixed methods | Kenya |  |
| daCruz | 2021 | Comprehensive approach to HIV/AIDS testing and linkage to treatment among men who have sex with men in Curitiba, Brazil | | Cross-sectional | Brazil |  |
| Dakum | 2021 | Effect of community antiretroviral therapy on treatment outcomes among stable antiretroviral therapy patients in Nigeria: A quasi experimental study | | Quasi-experimental | Nigeria |  |
| Dickson Gomez | 2021 | Cumulative Effects of Adding a Small Group Intervention to Social Network Testing on HIV Testing Rates Among Crack Users in San Salvador, El Salvador | | Program evaluation | El Salvador |  |
| Didiya | 2021 | Community led testing among people who inject drugs: A community centered model to find new cases of HIV and Hepatitis C in Nepal | | Mixed methods | Nepal |  |
| Donnell | 2021 | Incorporating oral PrEP into standard prevention services for South African women: a nested interrupted time-series study | | Quasi-experimental | South Africa |  |
| Dougherty | 2021 | Improving Services for HIV-Exposed Infants in Zambia and Cameroon Using a Quality Improvement Collaborative Approach | | Quasi-experimental | Cameroon, Zambia |  |
| Ferreyra | 2021 | Evaluation of a community-based HIV test and start program in a conflict affected rural area of Yambio County, South Sudan | | Program evaluation | South Sudan |  |
| Goodrich | 2021 | Development, Assessment, and Outcomes of a Community-Based Model of Antiretroviral Care in Western Kenya Through a Cluster-Randomized Control Trial | | Randomized controlled | Kenya |  |
| Grande | 2021 | Intensified Assisted Partner Notification Implementation in Botswana Increased Partner Identification but Not HIV Case-Finding: Findings Highlight the Need for Improved Data Monitoring | | Cohort | Botswana |  |
| Grasso | 2021 | Improving the benefits of HIV testing and referrals in large household surveys through active linkages to care: lessons and recommendations from the Namibia population-based HIV impact assessment (NAMPHIA), 2017 | | Cross-sectional | Namibia |  |
| Hodges | 2021 | Implementation of a Mobile Health Strategy to Improve Linkage to and Engagement with HIV Care for People Living with HIV, Tuberculosis, and Substance Use in Irkutsk, Siberia | | Cohort | Russia |  |
| Hoffman | 2021 | Multi-month dispensing of up to six months of antiretroviral therapy in Malawi and Zambia: Results of a closer-randomized trial. (under review) INTERVAL study | | Randomized controlled | Malawi, Zambia |  |
| Ibiloye | 2021 | Community health worker-led ART delivery improved scheduled antiretroviral drug refill among men who have sex with men in Lagos State, Nigeria | | Cohort | Nigeria |  |
| Indravudh | 2021 | Effect of community-led delivery of HIV self-testing on HIV testing and antiretroviral therapy initiation in Malawi: A cluster randomised trial | | Randomized controlled | Malawi |  |
| Indravudh | 2021 | Effect of door-to-door distribution of HIV self-testing kits on HIV testing and antiretroviral therapy initiation: a cluster randomised trial in Malawi | | Randomized controlled | Malawi |  |
| Kebaya | 2021 | Efficacy of Mobile phone use on adherence to Nevirapine prophylaxis and retention in care among the HIV-exposed infants in prevention of mother to child transmission of HIV: a randomized controlled trial | | Randomized controlled | Kenya |  |
| Kinuthia | 2021 | SMS messaging to improve retention and viral suppression in prevention of mother-to-child HIV transmission (PMTCT) programs in Kenya: A 3-arm randomized clinical trial | | Randomized controlled | Kenya |  |
| Kiyaga | 2021 | Uganda’s “EID Systems Strengthening” model produces significant gains in testing, linkage, and retention of HIV-exposed and infected infants: An impact evaluation | | Cohort | Uganda |  |
| Kumar | 2021 | Improving HIV Self‐Testing Social Network Interventions: The Role of Sexual Behavior Disclosure Among Chinese Men Who Have Sex with Men | | Quasi-experimental | People's Republic of China |  |
| Loch | 2021 | Improving the continuum of care monitoring in Brazilian HIV healthcare services: An implementation science approach | | Mixed methods | Brazil |  |
| Maatouk | 2021 | Community-led HIV self-testing for men who have sex with men in Lebanon: lessons learned and impact of COVID-19 | | Program evaluation | Lebanon |  |
| Macis | 2021 | Using Incentives and Nudging to Improve Non‑Targeted HIV Testing in Ecuador: A Randomized Trial | | Randomized controlled | Ecuador |  |
| Mackellar | 2021 | Annual home-based HIV testing in the Chokwe HealthDemographic Surveillance System, Mozambique, 2014 to 2019:serial population-based survey evaluation | | Cross-sectional | Mozambique |  |
| Magidson | 2021 | Project Khanya: results from a pilot randomized type 1 hybrid effectiveness-implementation trial of a peer-delivered behavioural intervention for ART adherence and substance use in HIV care in South Africa | | Randomized controlled | South Africa |  |
| Maruyama | 2021 | Bringing HIV services to key populations and their communities in Tanzania: from pilot to scale | | Program evaluation | Tanzania |  |
| Maughan-Brown | 2021 | Poor rates of linkage to HIV care and uptake of treatment after home-based HIV testing among newly diagnosed 15-to-49 year- old men and women in a high HIV prevalence setting in South Africa | | Cross-sectional | South Africa |  |
| Mbithi | 2021 | Assessing the Real-Time Impact of COVID-19 on TB and HIV Services: The Experience and Response from Selected Health Facilities in Nairobi, Kenya | | Cohort | Kenya |  |
| Mishra | 2021 | Systems Analysis to Increase HIV Testing Delivery and HIV Diagnosis in Primary Care Clinics in South Africa | | Program evaluation | South Africa |  |
| Muchedzi | 2021 | High HIV Positivity Rates Following Large-Scale HIV Self-Testing Implementation in Zimbabwe, 2018–2020 | | Program evaluation | Zimbabwe |  |
| Muhindo | 2021 | Text message reminders and peer education increase HIV and Syphilis testing among female sex workers: a pilot quasiexperimental study in Uganda | | Quasi-experimental | Uganda |  |
| Mulwa | 2021 | Reaching early adolescents with a complex intervention for HIV prevention: findings from a cohort study to evaluate DREAMS in two informal settlements in Nairobi, Kenya | | Cohort | Kenya |  |
| Nalwanga | 2021 | Utilization of a mobile phone application to increase access to sexual and reproductive health information, goods, and services among university students in Uganda | | Randomized controlled | Uganda |  |
| Ndhlovu | 2021 | The transient effect of a peer support intervention to improve adherence among adolescents and young adults failing antiretroviral therapy in Harare, Zimbabwe: a randomized control trial | | Randomized controlled | Zimbabwe |  |
| Neuman | 2021 | Does community-based distribution of HIV self-tests increase uptake of HIV testing? Results of pair-matched cluster randomised trial in Zambia | | Randomized controlled | Zambia |  |
| Nichols | 2021 | Community-based delivery of HIV treatment in Zambia: costs and outcomes | | Cohort | Zambia |  |
| Njuguna | 2021 | Financial Incentives to Increase Pediatric HIV Testing: a Randomized Trial | | Randomized controlled | Kenya |  |
| Okoboi | 2021 | Cost-Effectiveness of Peer-Delivered HIV Self-Tests for MSM in Uganda | | Economic evaluation | Uganda |  |
| Okoko | 2021 | Improvements in pediatric and adolescent HIV testing and identification in western Kenya under the Accelerating Children’s HIV/AIDS Treatment initiative | | Program evaluation | Kenya |  |
| Pines | 2021 | A comparison of the effectiveness of respondent‐driven and venue‐based sampling for identifying undiagnosed HIV infection among cisgender men who have sex with men and transgender women in Tijuana, Mexico | | Cross-sectional | Mexico |  |
| Sanchez | 2021 | Toward improved adherence: a text message intervention in an human immunodeficiency virus pediatric clinic in Guatemala City | | Randomized controlled | Guatemala |  |
| Sekiziyivu | 2021 | Task Shifting for Initiation and Monitoring of Antiretroviral Therapy for HIV-Infected Adults in Uganda: The SHARE Trial | | Randomized controlled | Uganda |  |
| Settergren | 2021 | Importance of support groups to the health and well-being of vulnerable children and young people living with HIV: a case study of the Kids Clubs program in Haiti | | Mixed methods | Haiti |  |
| Shahmanesh | 2021 | Effect of peer-distributed HIV self-test kits on demand for biomedical HIV prevention in rural KwaZulu-Natal, South Africa: a three-armed cluster-randomised trial comparing social networks versus direct delivery | | Randomized controlled | South Africa |  |
| Sibanda | 2021 | Comparison of community-led distribution of HIV self-tests kits with distribution by paid distributors: a cluster randomised trial in rural Zimbabwean communities | | Randomized controlled | Zimbabwe |  |
| Stonbraker | 2021 | Clinician Use of HIV-Related Infographics During Clinic Visits in the Dominican Republic is Associated with Lower Viral Load and Other Improvements in Health Outcomes | | Quasi-experimental | Dominican Republic |  |
| Sumari-de Boer | 2021 | Effect of Digital Adherence Tools on Adherence to Antiretroviral Treatment Among Adults Living With HIV in Kilimanjaro, Tanzania: A Randomized Controlled Trial | | Randomized controlled | Tanzania |  |
| Suryana | 2021 | The Impact of Universal Test and Treat Program on Highly Active Anti Retroviral Therapy Outcomes (Coverage, Adherence and Lost to Follow Up) at Wangaya Hospital in Denpasar, Bali-Indonesia: A Retrospective Cohort Study | | Cohort | Indonesia |  |
| Taiwo | 2021 | Effect of Text Messaging Plus Peer Navigation on Viral Suppression Among Youth With HIV in the iCARE Nigeria Pilot Study | | Cohort | Nigeria |  |
| Tesfaye | 2021 | Effect of the Test and Treat Strategy on Mortality Among HIV-Positive Adult Clients on Antiretroviral Treatment in Public Hospitals of Addis Ababa, Ethiopia | | Cohort | Ethiopia |  |
| Wang | 2021 | Feasibility and impact of near-point-of-care integrated tuberculosis/HIV testing in Malawi and Zimbabwe | | Quasi-experimental | Malawi, Zimbabwe |  |
| Wechsberg | 2021 | Outcomes of Implementing in the Real World the Women’s Health CoOp Intervention in Cape Town, South Africa | | Randomized controlled | South Africa |  |
| Widyanthini | 2021 | HIV self-testing for men who have sex with men: an implementation trial in Indonesia | | Cohort | Indonesia |  |
| Wirth | 2021 | Population uptake of HIV testing, treatment, viral suppression, and male circumcision in Botswana: a cluster-randomized trial | | Randomized controlled | Botswana |  |
| Wu | 2021 | Social Media–Based Secondary Distribution of Human Immunodeficiency Virus/Syphilis Self-testing Among Chinese Men Who Have Sex with Men | | Cohort | People's Republic of China |  |
| Yang | 2021 | Sexual Health Influencer Distribution of HIV/Syphilis Self-Tests Among Men Who Have Sex With Men in China: Secondary Analysis to Inform Community-Based Interventions | | Cohort | People's Republic of China |  |
| Ybarra | 2021 | A Pilot RCT Evaluating InThistoGether, an mHealth HIV Prevention Program for Ugandan Youth | | Randomized controlled | Uganda |  |
| Yun | 2021 | Mobile Phone Intervention Based on an HIV Risk Prediction Tool for HIV Prevention Among Men Who Have Sex With Men in China: Randomized Controlled Trial | | Randomized controlled | People's Republic of China |  |
